# Engineered nanoconjugates for simultaneous detection and degradation of stroke-associated microthrombi

**DOI:** 10.7150/thno.119705

**Published:** 2026-01-01

**Authors:** Audrey Picot, Charlène Jacqmarcq, Célia Seillier, Sara Martinez de Lizarrondo, Maxime Gauberti, Ankita Talukdar, Igor Khalin, Clarisse Mouriaux, Pierre Mangin, Didier Goux, Peter Schmidt, Denis Vivien, Thomas Bonnard

**Affiliations:** 1 Normandie Université, UNICAEN, INSERM, PhIND (Physiopathology and Imaging of Neurological Disorders) Institut Blood and Brain @ Caen-Normandie, Cyceron, Caen, France.; 2 CHU CAEN, Clinical Research Department, CHU de Caen côte de Nacre, Caen, France.; 3 CHU CAEN, Radiology Department, CHU de Caen côte de Nacre, Caen, France.; 4 Normandie Université, UNICAEN, Université Caen Normandie, US EMerode, CMAbio3: Centre de Microscopie Appliquée à la Biologie, Caen, France.; 5 Institute for Stroke and Dementia Research, LMU University Hospital, 81377, Munich, Germany.; 6 Université de Strasbourg, INSERM, EFS Grand Est, BPPS UMR-S1255, FMTS, 67065 Strasbourg, France.; 7 CSL Ltd., Bio21 Institute, Parkville, Victoria 3010, Australia.

**Keywords:** ischemic stroke, microthrombi, iron oxide microparticles, polydopamine, theranostic

## Abstract

**Background:**

Microthrombi obstructing downstream microcirculation in acute ischemic stroke (AIS) are difficult to treat and visualize with current imaging methods.

**Methods:**

To address this need, a novel theranostic agent, IO@PDA@tPA, was developed by combining iron oxide microparticles (IO) coated with polydopamine (PDA) and conjugated with recombinant tissue-type plasminogen activator (r-tPA). The amidolytic and fibrinolytic capacities of r-tPA grafted on IO@PDA were assessed using the spectrofluorometric test, the clot lysis assay, and the whole blood halo assay. IO@PDA@tPA was then tested *in vivo* in a preclinical ischemic stroke model induced by thrombin injection into the middle cerebral artery in both non-diabetic and diabetic mice. Two doses equivalent to 2.5 and 5 mg/kg r-tPA were tested. The presence of microthrombi was monitored via molecular MRI. A series of *T*_2_*-weighted sequences for microthrombi imaging and magnetic resonance angiography (MRA) was performed over 45 min. At 24 h, lesion size, vessel patency, and hemorrhagic transformation were assessed with *T_2_*-weighted imaging, MRA, and T_2_^*^-weighted MRI, respectively. A grip test was performed to assess functional recovery one day before stroke (baseline), and at 24 h and five days after stroke. Additionally, inflammatory processes were evaluated five days post-stroke by flow cytometry in the non-diabetic cohort.

**Results:**

This agent exhibited *in vitro* clot lysis activity. *In vivo*, administration of IO@PDA@tPA at one-quarter of the standard r-tPA dose enabled both visualization and degradation of microthrombi, as detected by T_2_^*^-weighted MRI. This treatment significantly reduced lesion size and promoted recanalization 24 h after stroke onset. In the hyperglycemic mice cohort, the agent demonstrated efficacy comparable to r-tPA without increasing hemorrhagic risk—a common complication of free r-tPA. Moreover, full functional recovery observed within five days post-stroke. Flow cytometry indicated that IO@PDA@tPA mitigated inflammatory processes.

**Conclusion:**

IO@PDA@tPA represents a promising theranostic agent targeting microthrombi in AIS, reducing the required r-tPA dose and limiting associated side effects.

## Introduction

Acute Ischemic Stroke (AIS) is defined by the formation of a blood clot in the cerebral vasculature. It is the leading cause of acquired disability in adults and the second leading cause of dementia worldwide 1. Thrombolysis, with the intravenous administration of recombinant tissue-type plasminogen activator (r-tPA) Alteplase or its mutant Tenecteplase combined with or without mechanical thrombectomy, are the only available treatments for AIS. Both treatments aim to remove the clot from the obstructed vessel to restore its patency and enable blood reperfusion within the infarcted brain area. Unfortunately, even if the recanalization is complete, the downstream microcirculation may remain obstructed by microthrombi 2. The persistence of these microthrombi contributes to ongoing ischemia in the affected brain regions, exacerbating the initial damage and leading to a poorer recovery prognosis 3. Microthrombi can originate from the disintegration and migration of the proximal clot or from the secondary formation of microvascular thrombosis 2,4,5.

A retrospective analysis from MR CLEAN trial revealed that 9 to 22% of patients undergoing mechanical thrombectomy suffer from incomplete microvascular reperfusion that may be attributed to clot fragmentation and migration into vessels distal to the main occlusion 6,7. In addition, patients who exhibit microthrombi after successful recanalization of arteries are more at risk of experiencing cognitive decline and dementia 8. This correlation between microthrombi and worse functional prognosis emphasizes the urge of addressing microthrombi as a critical therapeutic target to improve long-term outcomes in AIS patients. The limited management of microthrombi is partly due to diagnostic challenges. Despite advances in brain imaging techniques, such as diffusion-weighted magnetic resonance imaging (MRI) 9 and transcranial Doppler, microthrombi remain difficult to detect due to their small size, especially during the AIS. Current imaging modalities often lack the resolution needed to visualize these microvascular obstructions accurately.

The efficacy of current AIS treatments is further constrained by their narrow therapeutic window—4.5 h for thrombolysis and up to 24 h for thrombectomy 10—drastically reducing the number of patients eligible for these interventions. As a result, over 80% of IS patients do not benefit from thrombolysis either because of the short therapeutic window or the comorbidities factors that weigh negatively on the r-tPA benefit-risk ratio 11. Comorbidities such as hypertension, diabetes, and atrial fibrillation complicate the administration of r-tPA, increasing the risks of adverse outcomes, including hemorrhagic events 11. Thrombolysis is also associated with secondary effects, including inflammation, increased blood-brain barrier permeability, and hemorrhagic transformation (HT), particularly in diabetic patients 12,13. The inflammatory response triggered by thrombolysis can exacerbate brain tissue damage, while the increased permeability of the blood-brain barrier heightens the risk of hemorrhage 14. Indeed, r-tPA treatment can induce intracerebral hemorrhage in up to 7% of patients 15. This significant risk underscores the need for more targeted therapeutic strategies that minimize systemic exposure to r-tPA, thereby reducing the likelihood of hemorrhagic complications.

Our group recently developed a contrast agent composed of commercial iron oxide nanoparticles clusterized with polydopamine, capable of detecting microthrombi in the infarct cortex using MRI in a preclinical mouse model of AIS 16. We showed that the polydopamine matrix enables the specific targeting of microthrombi *via* the formation of a protein corona on its surface in the bloodstream. Its composition includes proteins involved in the acute coagulation phase such as fibrinogen 16. Here, we produced iron oxide nanocrystals using a co-precipitation method, which were self-assembled *via* polydopamine polymerization into 600-nanometers particles (IO@PDA). We grafted r-tPA on the surface of the particles and developed a microthrombi specific theranostic tool. This targeted delivery approach of r-tPA directly to the edge of the clots enabled a reduction of the effective dose and thereby a reduction of r-tPA-associated deleterious effects. The IO@PDA@tPA microthrombi theranostic tool was tested in an AIS mouse model in condition of comorbidity, comparing non-diabetic and diabetic cohorts. This theranostic approach offers a more precise, efficient, and safer procedure for delivering an established thrombolytic therapy whilst allowing to monitor microthrombi degradation via MRI, potentially extending the therapeutic window, and reducing the incidence of HT.

## Materials and Methods

### IO@PDA synthesis

The IO@PDA are synthetized by coprecipitation in basic conditions and subsequently clusterized via dopamine polymerization into polydopamine. Full protocol was previously described 20.

### Determination of iron concentration

The iron concentration of IO@PDA suspensions was determined with a colorimetric protocol (FerroZine Iron Reagent, Sigma-Aldrich). Complete protocol was previously described 20.

### Determination of the hydrodynamic diameter of the particles

The hydrodynamic diameter and polydispersity index (PDI) was measured by Dynamic light scattering (DLS) with a Nano ZS (Malvern Instruments, Worcestershire, UK). Sample were diluted in H2O and experiment were performed at 25 °C. All IO@PDA batches were measured around 600 nm, in triplicate.

### IO@PDA@tPA synthesis

#### Dialyzed r-tPA

Commercial recombinant tPA (Actilyse®, Boehringer Ingelheim) was reconstituted in ultra-pure water and transferred into a dialysis membrane (Spectra/Por® Dialysis Membrane, MWCO 12-14.000, TRIAL KITS). The dialysis membrane filled with r-tPA was placed under continuous agitation in HEPES buffer, pH 7.4 for 48 h at 4 °C followed by concentration measurement using a Nanodrop (NanoPhotometer® N50, IMPLEN; MW = 69 000 g.mol; ε = 107620 l/g*cm).

#### Conjugating r-tPA on IO@PDA

500 µL dialyzed r-tPA at 2 mg/mL are mixed with 500 µL of IO@PDA at 1 mg/mL iron concentration resulting in a final concentration of 1 mg/mL r-tPA and 0.5 mg/mL of iron oxide (IO). The mixed solution is incubated at 4 °C for 1 h under constant rotation. After 1 h, a magnet was used to collect the r-tPA-conjugated IO@PDA. The particles were resuspended in 0.3 M mannitol solution. Remaining un-bound r-tPA in solution was quantified in triplicate using nanodrop (MW = 69000 g.mol; ε = 107620 l/g*cm). The amount of IO@PDA conjugated with r-tPA was calculated by subtracting free r-tPA from the initial concentration. The IO@PDA@tPA are stored at 4 °C under constant agitation until further used.

#### Transmission electron microscopy

For characterisation of IO@PDA or IO@PDA@tPA, we deposited a drop of particles in suspensions that we hydrophilized on a grid made from carbon and formvar 200-mesh. Images of IO@PDA@tPA were acquired using a digital camera (GATAN, ORIUS 200) installed on a transmission electron microscope 1011 (JEOL). The treatment of the particle suspensions and the image capture were done at the CMAbio3 center (Centre de Microscopie Appliquée à la Biologie, Biological Microscopy Facility Center UFR des Sciences, US EMerode, University of Caen Normandy).

### Verifying enzymatic and fibrinolytic activity of IO@PDA-coupled r-tPA

#### Spectrofluor test

Several dilutions of r-tPA alone and IO@PDA@tPA in a Tris solution (50 mM Tris (pH 8.0) containing 150 mM NaCl) were made. Based on the data sheet, 500 ng, 250 ng, and 100 ng were the most suitable. Then, the various samples were incubated (in triplicates) in the presence of a fluorogenic substrate (5 mM, Spectrofluor FL444). The reaction was carried out at 37 °C in a total volume of 100 µL. The amidolytic activity was measured as the change in fluorescence emission at 440 nm (excitation at 360 nm) over time with a multimode microplate reader (Spark®, TECAN). Amidolytic activity of r-tPA was determined by measuring the fluorescence increase over time (V0) using a spectrofluorometric assay, and the initial rates were plotted relative to the amount of protein mass added to the reaction.

#### Clot lysis test

The effect of IO@PDA@tPA during clot formation and lysis was studied by monitoring the change in turbidity in human plasma using a microplate reader (FLUOstar Optima, BMG Labtech). Calcium chloride (final concentration, 25 mM) was added to citrated plasma and diluted in the same volume of a 10 mM HEPES buffer supplemented with 4 mg/mL bovine serum albumin (BSA) adjusted to pH 7.4 in order to promote coagulation. Samples were incubated at 37 °C with IO@PDA, r-tPA, IO@PDA@tPA or IO@PDA + r-tPA at 1 and 5 nM r-tPA equivalence, and absorbance (405 nm) was monitored for 12 h every 30 s at 37 °C. Results are expressed as the time to achieve 75% maximal absorbance (clotting time (CT)), and the 50% lysis time (LT) was calculated as the time from initiation of clot formation to the time at which maximal absorbance fell to 50%. All experiments were performed in triplicate.

#### Halo assay

The blood samples anticoagulated with citrate were obtained from healthy donors through the local blood bank, the Etablissement Français du Sang (EFS, Caen, France). The collection and transfer of these samples were conducted in accordance with the “agreement for the transfer of products derived from blood or its components for non-therapeutic purposes n° PLER /2021/005” established between the EFS Hauts-de-France Normandie and the Institut National de la Santé et de la Recherche Médicale (INSERM). This agreement guarantees compliance with best practices for venipuncture and the ethical use of blood from healthy volunteers in research, including the acquisition of informed consent from participants. As all EFS activities meet ethical standards, this agreement did not require review by an institutional review board. Halo assay *in vitro* thrombolysis test was performed following a published protocol 21. Droplets of the clotting mixture (Innovin (Dade® Innovin®, Siemens, Munich, Germany), CaCl_2_ 0.25M, HEPES buffer (25 mM HEPES, 137 mM NaCl, pH 7.4)) were deposited on the bottom edge of the wells of a 96 well plate. The clotting mixture is spread around the edge of the wells with the tip of a P100 micropipette containing 20 µL of blood. The blood is slowly released and mixed around the edge of the well thereby making use of the fluidic cohesion effect. After incubation at 37 °C for 30 min, the clots should have a homogeneous halo shape at the bottom of the wells, leaving the center area of the well clear and empty. 2 conditions were tested: r-tPA and IO@PDA@tPA at 0.25; 0.1 and 0.075 mg/mL (r-tPA equivalence). The fibrinolysis rate was monitored by measuring absorbance at 510 nm every minute for 2 h at 37 °C using a multimode microplate reader (Spark®, TECAN). Negative controls were prepared by adding 70 μL of phosphate buffer saline (PBS) to halo thrombi in the absence of any fibrinolytic agent, while positive controls consisted of a well containing 25 μL of blood mixed with 75 μL of PBS (without the clotting mixture). The absorbance values from positive control wells represented complete degradation (A_total_), whereas those from negative control corresponded to no degradation (A_zero_). The percentage of degradation at each time point was then calculated using the following formula: D_x_(t) = 100*((A_x_(t) - A_zero_(t))/(A_total_(t) - A_zero_(t))). The assay allows to determine maximum degradation (D_max_), the activation time (A_t_) and the maximum clot lysis rate (CLR_max)_).

#### *In vitro* flow-based assays

The first model involves forming fibrin without platelet aggregates. PDMS microfluidic chambers (0.09 x 1 mm) were coated with tissue factor (12 mM) for 1 h at room temperature (RT). Non-specific adhesion was prevented with a human serum albumin solution (10 mg/mL) in phosphate buffered saline for 30 min at RT. To form fibrin, citrated (0.38%) platelet-poor plasma (PPP) was recalcified by adding 20 mM of CaCl_2_ and immediately perfused at 500 s^-1^. Fibrin formation was stopped by perfusing non-recalcified PPP before perfusing PPP containing IO@PDA, r-tPA (1 µg/mL), or IO@PDA@tPA at 500 s^-1^ for 6 min.

The second model involves forming fibrin-rich thrombi. PDMS microfluidic chambers (0.09 x 1 mm) were coated with type I fibrillar collagen (HORM collagen, Takeda, 200 µg/mL) for 1 h at RT and then with tissue factor (Thromborel S, Siemens Healthineers, 12 nM) for 30 min at RT. Non-specific adhesion was prevented with a human serum albumin solution (10 mg/mL) in phosphate buffered saline for 30 min at RT. To form fibrin-rich thrombi, citrated (0.32%) human whole blood was recalcified with CaCl_2_ (12.5 mM) and MgCl_2_ (3.5 mM) immediately before perfusing it through coated channels at 1500 s^-1^. A washing step of the fibrin-rich thrombi was performed before perfusing PPP obtained from the same donor in presence of IO@PDA, r-tPA (1 µg/mL), or IO@PDA@tPA at 1500 s^-1^ for 6 min.

Fibrin and fibrin-rich thrombi were observed in real time by differential interference contrast technique using an inverted Leica DMI4000 B microscope (Leica Microsystems) and a 40x, 1.25 numerical aperture oil objective. Images were acquired with complementary metal-oxide semi-conductor (CMOS) ORCA FLASH-4 LT camera (Hamamatsu Photonics, Hamamatsu, Japan) and analyzed using ImageJ software (National Institutes of Health, Bethesda, MD).

### Assessing the antioxidant effects of the IO@PDA with *in vitro* experiments

#### Primary neuronal cell culture

Primary cultures of cortical neurons were prepared from fetal mice (embryonic day 14). Briefly, cells were cultured on 24-well plates, previously coated with poly-D-lysine (0.1 mg/mL) and laminin (0.02 mg/mL), in Dulbecco's Modified Eagle Medium (DMEM) supplemented with 5% fetal bovine serum, 5% horse serum and 1 mM glutamine. Cultures were maintained at 37 °C in a humidified 5% CO_2_ atmosphere. Cytosine β-D-arabinoside (10 μM) was added after 3 days *in vitro* (DIV) to inhibit glial proliferation in the 24-well plates.

#### Oxygen and glucose deprivation

Neuronal cultures (12 DIV) were subjected for 1 h to OGD in a hypoxic chamber (AWEL International, Ruskinn InvivO_2_ 500) programmed at 0.2% O_2_, 5% CO_2_ and 37 °C in glucose serum-free deoxygenated DMEM. Cortical neurons are treated with IO@PDA at 0.05; 0.075; 0.1; and 0.25 mg/mL in the hypoxic chamber, at the beginning of OGD. For reoxygenation, D-glucose (4.5 mg/L) is added in cell media under normoxic conditions. Controls were subjected to sham washes.

#### Neuronal death

Neuronal death was quantified 4 h after reoxygenation by measuring the lactate dehydrogenase activity (LDH) released from damaged cells into the bathing medium with a cytotoxicity detection kit (Roche Diagnostics, 91963).

#### Reactive Oxygen Species Assay

Reactive Oxygen Species (ROS) formation was quantified 4 h after reoxygenation by using the DCFDA - Cellular ROS Assay Kit / Reactive Oxygen Species Assay Kit (ab113851). Briefly, cells were washed 2 times with 1X buffer provided by the kit and were stained for 45 min at 37 °C in the dark by adding diluted DCFDA (Dichlorodihydrofluorescein Diacetate solution). Cells were re-washed 2 times with 1X buffer provided by the kit and live cell microscopy was performed. At least 3 pictures were taken for each condition. Then ROS were counted to get the number of ROS per mm^2^, and compared between control (no IO@PDA and no OGD with IO@PDA) and OGD condition.

### *In vivo* experiments

#### Animals

All experiments were conducted in compliance with French ethical law (Decrees 2013-118 and 2020-274) and the European Communities Council guidelines (2010/63/EU). Experiments were approved by the local ethical committee of Normandy (CENOMEXA, APAFIS#36663). Animals were provided and maintained under specific pathogen-free conditions at the Centre Universitaire de Ressources Biologiques (CURB, Basse-Normandie, France), and all had free access to food and tap water. Mice were housed in a room where the temperature was adjusted with *ad libitum* water and food. All experiments were performed on 8-week-old male Swiss mice. A total of 136 mice were included, separated into 3 cohorts, 2 cohorts of normoglycemic mice pooled together and 1 cohort of hyperglycemic mice. Mice were randomly and blindly assigned to experimental groups, ensuring balanced representation from each cage. Intravenous injections of the contrast agent or treatments were performed *via* a tail vein catheter. After surgery, animals recovered in a warm cage before taking them back to the animal facility.

#### Hyperglycemic mice model induced by streptozotocin (STZ)

STZ (S0130_500MG, Sigma-Aldrich) was injected intraperitoneally to the mice during five days at 40 mg/kg. Blood sugar was monitored at 1-day pre-treatment, 1 day, 2 days, 8 days, 14 days and 21 days post treatment after 4 h of fasting. On day 21, mice were included in the study if the blood sugar was above 200 mg/dl.

#### Thrombin model

Based on our previous paper 17, the procedure was performed as follows: before the surgical procedure, a pipette was made with hematologic micropipettes (calibrated at 15 mm/L; Assistent ref. 555/5; Hoecht, Sondheim-Rhoen, Germany) by using an electrophysiology puller (PC-10; Narishige). Thereafter, the micropipette was pneumatically filled with 1 µL of purified murine alpha-thrombin (approximately 1000 NIH units/mg; Sigma-Aldrich) by applying negative pressure. Mice were anesthetized with isoflurane (5%, 70/30 NO_2_/O_2_). Mice were placed in a stereotaxic device and maintained under anaesthesia with isoflurane (1.5-2%, 70/30 NO_2_/O_2_) at 37 °C by the integrated heat animal holder. Before beginning the surgery, buprenorphine (BUPRECARE, H0270. 54000561, 0.3 mg/mL) was injected for analgesia. An incision was then performed on the right side between the ear and the eye, and the temporal muscle was removed. After craniotomy window, the dura was removed slowly, and the middle cerebral artery (MCA) could be identified. The pipette was inserted into the MCA and 1 µL of alpha-thrombin (0.75 UI) was pneumatically injected (by applying positive pressure with a syringe connected to the pipette through a catheter) to induce the formation of a clot *in situ*. The pipette was removed 5 min after the injection of alpha thrombin at which time the clot had stabilized.

#### IO@PDA@tPA preparation and injection

As described before, r-tPA is conjugated to the particles 24 h before inducing the thrombin stroke model on mice. Based on the deduced r-tPA concentration conjugated to IO@PDA and the animal weight, the intravenous injection volume of IO@PDA@tPA in mice at the desired doses of r-tPA is determined. For example, **to inject 2.5 mg/kg r-tPA equivalence** of IO@PDA@tPA to a 40 g mouse: **2.5** (concentration desired) x **40** (mouse weight) / **0.5** (r-tPA concentration fixed on the particles) = **200µL**.

#### MRI acquisition

Experiments were performed using a 7-T TEP-MRI scanner using a volume coil resonator (Bruker, Germany). Mice were anesthetized with isoflurane (1.5 to 2.0%) and maintained at 37 °C by the integrated heat animal holder, and the breathing rate was monitored during the imaging procedure.

Brain scans including an alternation of T_2_*-weighted sequence for iron-sensitive imaging with TR/TE 50ms /8.6 ms and TOF sequence to visualize vascular structure, with TR/TE = 12 ms/4.2 ms were made 20 min after treatments' injection : IO@PDA (2 mg/kg); IO@PDA@tPA at 2.5 mg/kg r-tPA and 2 mg/kg IO@PDA equivalence; IO@PDA@tPA at 5 mg/kg r-tPA and 4 mg/kg IO@PDA equivalence; IO@PDA (2 mg/kg) + r-tPA (2.5 mg/kg) for 45 min.

Then brain scans including *T_2_*-weighted (Rapid Acquisition with Relaxation enhancement (RARE) sequence, with TR/TE = 3500 ms/40 ms), T_2_*-weighted sequences (fast-low angle shot (FLASH) sequence, with TR/TE = 50 ms/3.5 ms) and time-of-flight (TOF) sequences (TR/TE = 12 ms/4.2 ms) were made 24 h after treatments' injection to visualize the lesion size, the recanalization and hemorrhagic transformation.

A *T_1_*-weighted RARE sequence was performed to obtain whole-body images (TE/TR 9/1200 ms, with 70 × 70 × 500 μm^3^ spatial resolution).

Fast acquisition of the brain for pharmacokinetics study was performed using a FLASH sequence with only one slice and TR/TE 20 ms/7 ms with a flip angle of 8°. The quantification of negative signal detected on 3D T_2_*-weighted images, and 3D representation of IO@PDA or IO@PDA@tPA were done *via* segmentation and using the thresholding module of Slicer software (v4.11). Results are presented as the area or volume of signal void (in mm^2^ or mm^3^ respectively).

#### Two-photon microscopy

Two-photon images were scanned using Bruker Ultima 2P Plus (Billerica, Massachusetts, USA). The glass slide containing coronal section of the mouse brain was placed onto custom-made platform under an upright microscope. Image acquisition was performed using laser (Chameleon Vision II) from 917 Coherent (Glasgow, Scotland) with an excitation wavelength of 920 nm and power of 300 Pockels and objective XLUMPlanFL N 20×/1.0 W (Olympus, Tokyo, Japan). PMT detectors with red, green and blue filters with master gain 500 were used. Images were taken as 50 μm deep Z-stacks (Galvo, dwell time 3.6, 1024 × 1024 (2048x2048 for zoomed), 13 bit).

#### Grip test

Functional recovery was assessed by measuring the animal's paws strength with a BIO-GS3 Grip Strength Test (Bioseb). Comparison between the baseline (day before stroke) and 1 day and 5 days after stroke for both paws and a ratio between the strength of the left paw and the right was made.

#### Corridor test

To assess sensorimotor function, we designed a custom-made corridor task, based on the ability of animals to explore objects. The apparatus consisted of a black PVC squared corridor (120 cm long, 6 cm wide, 16 cm wall's height). On each wall (left and right) four objects (custom-made, 3D-printed, 2 cm height, 2 width, 1 cm depth), spaced 16.5 cm apart, were fixed at 1 cm from the ground. Shape and colour of the objects were changed on each test day. Mice were habituated for 30 min in the experiment room. Then each mouse was placed at the beginning of the corridor, the head facing the end, and allowed to cross freely through the corridor during 1 min period. Exploration of the objects was quantified on each side of the corridor.

### *Ex vivo* experiments

#### Flow cytometry

Five days after the induction of ischemic stroke, mice were euthanized and perfused intracardially with heparinized PBS (15 U/mL). Brains were carefully extracted and processed using the Adult Brain Dissociation Kit (Miltenyi Biotec) according to the manufacturer's instructions. Tissue dissociation was performed using the gentleMACS™ Octo Dissociator with Heaters (Miltenyi Biotec). After enzymatic and mechanical dissociation, single-cell suspensions were obtained and filtered through a 70 µm cell strainer. Cells were then stained with fluorochrome-conjugated monoclonal antibodies targeting surface markers for 15 min in the dark at 4 °C. Two antibody panels were used: (1) Myeloid cell panel: Leukocytes were identified using the CD45 (PE-Cy7, 30-F11, BD Biosciences). Among leukocytes, myeloid cells were gated as CD11b⁺ (VioBlue, REA592 130-113-810, Miltenyi Biotec), from which microglia and neutrophils were discriminated using the Ly6G (FITC, 1A8, BD Biosciences). CNS-associated macrophages (CAMs) were further defined by the use of the CD206 (APC, C068C2, BioLegend) within the CD45⁺/CD11b⁺ population. (2) T lymphocyte panel: T lymphocytes were identified using the CD3ε (Brilliant Violet 510, 145-2C11, BD Biosciences), and CD4⁺ and CD8⁺ subsets were distinguished using CD4 (APC, RM4-5, BD Biosciences), and CD8α (PE-Cy7, 53-6.7, BD Biosciences) respectively. Flow cytometry was performed on a FACSVerse cytometer (BD Biosciences), and data were analyzed using FlowJo software (v7.6.5, TreeStar Inc.).

### Statistical analysis

The data are reported as mean ± Standard Deviation (SD). Statistical analyses were performed in a blinded manner using GraphPad Prism software (version 8.0). The normality of the data distribution was assessed using the Shapiro-Wilk test. A two-way ANOVA or Kruskal-Wallis with Tukey's multiple comparisons test was used when more than two groups were compared. Differences were considered statistically significant when the p-value was < 0.05.

### Acceptance criteria

Mice were excluded in case of death during the experimental procedure, technical problems, or hemorrhage, or if mice presented a lesion size superior to 35 mm² or inferior to 10 mm² (without injection of a thrombolytic agent).

## Results/Discussion

### IO@PDA@tPA: synthesis and characterization *in vitro*

**Figure 1:**
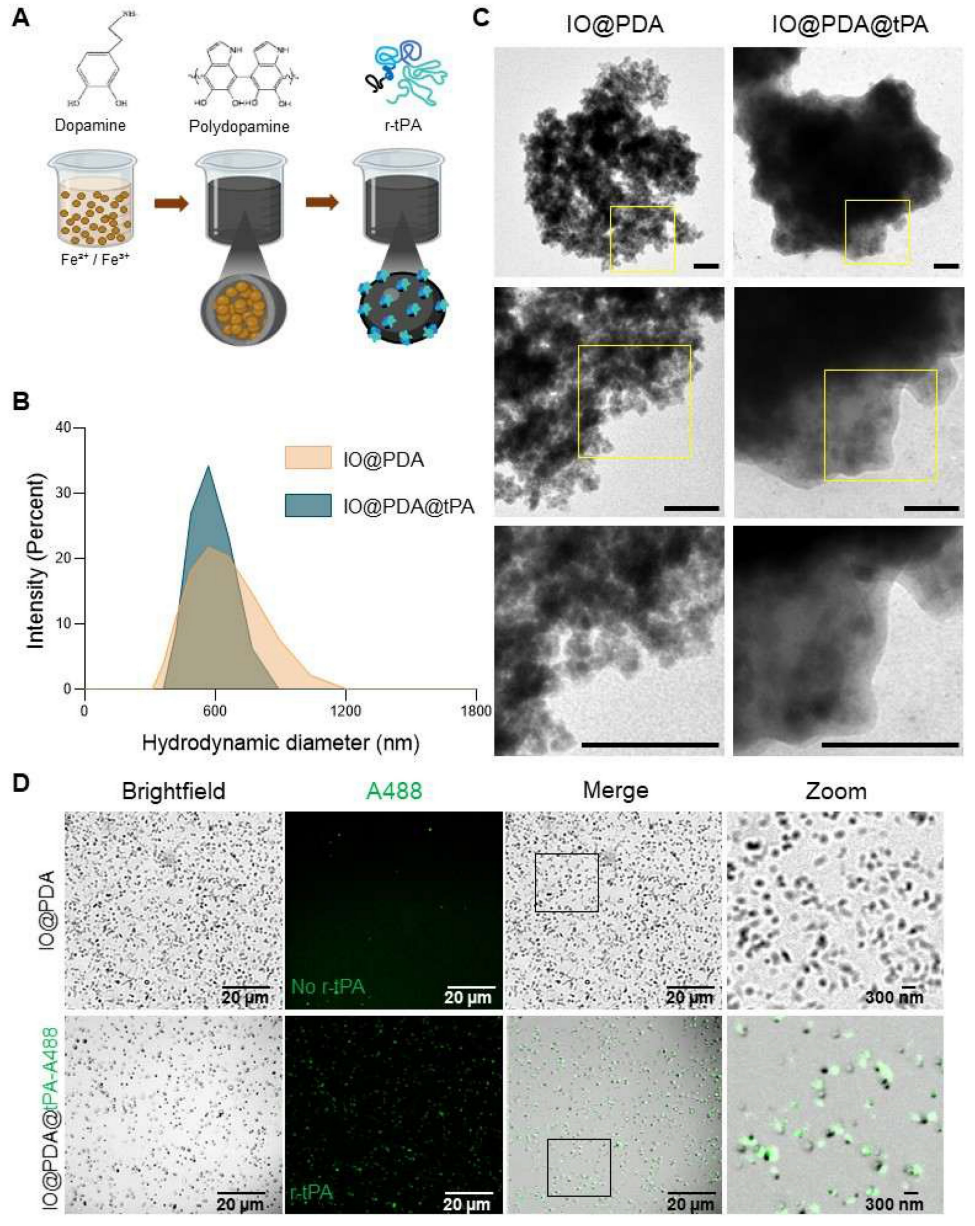
** Synthesis and characterization of the IO@PDA@tPA.** Schematic illustration of the IO@PDA@tPA synthesis (A). DLS analysis of IO@PDA and IO@PDA@tPA, provided the mean hydrodynamic diameter (n = 3 particles preparations) (B). Transmission electron microscopy images of IO@PDA and IO@PDA@tPA (scale bar = 0.22 µm) (C). IO@PDA were functionalized with r-tPA grafted with an Alexa Fluor 488. Confocal microscopy confirmed the functionalization of the r-tPA (green) on the IO@PDA (darkfield) (D).

We synthesized iron oxide nanoparticles (IO) *via* a simple co-precipitation of Fe²⁺/Fe³⁺ ions, leading to nanocrystal formation. These nanocrystals were first coated with dopamine. Then, the polymerization of dopamine was induced in an alkaline buffer triggering the formation of a polydopamine matrix with trapped nanocrystals (IO@PDA; Figure 1A). The resulting IO@PDA particles had a hydrodynamic diameter of 614.6 nm ± 74.5 nm and a zeta potential of -23.79 mV ± 3.6 mV (Table 1, Figure 1B). Subsequently, a defined concentration of dialyzed r-tPA was added to the IO@PDA batch and incubated for 1 h at 4 °C under constant rotation, allowing the r-tPA to graft onto the particle surfaces (Figure 1A; Figure S1). The average diameter of the IO@PDA@tPA is slightly modified, a slight increase in particle size dispersion is observed, with no visible aggregation. Moreover, they retained the same zeta potential as the unmodified IO@PDA (Figure 1B-D). Transmission electron microscopy (TEM) of the IO@PDA@tPA revealed a surrounding veil structure (Figure 1C). Additionally, confocal microscopy using r-tPA conjugated with Alexa Fluor 488 confirmed successful r-tPA functionalization on the IO@PDA surface (Figure 1D).

**Figure 2:**
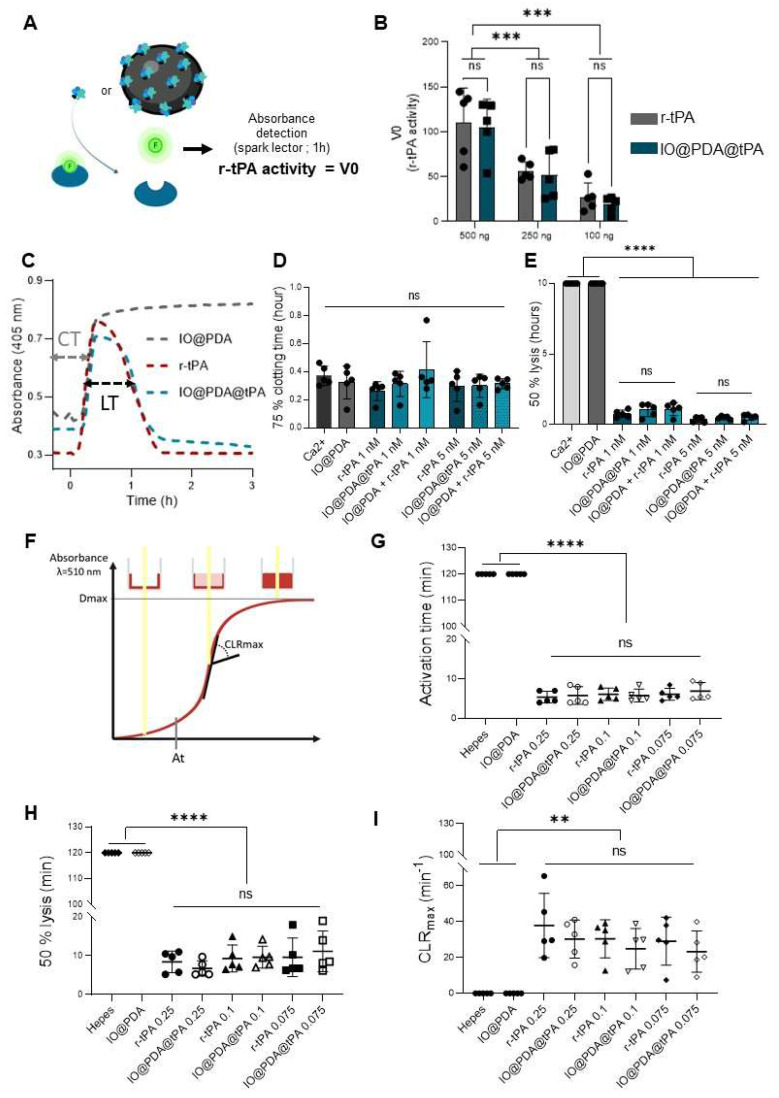
** r-tPA keeps its amidolytic and fibrinolytic capacities when functionalized on the IO@PDA.** Schematic principle of the spectrofluor test (A). The enzymatic activity test demonstrated that r-tPA kept its amidolytic properties when grafted on the IO@PDA (2-way ANOVA, multiple comparisons, n = 5 per group) (B). Representative curves acquired after the human plasma clot lysis exemplified how clotting time (CT) and lysis time (LT) were obtained (C). The human plasma clot lysis allowed the mean 75% clotting time (D) and the mean 50% clot lysis time (E) quantification in the presence of different concentrations of free r-tPA and IO@PDA@tPA. No significant difference between free or IO@PDA-grafted r-tPA were observed (2-way ANOVA, multiple comparisons, n = 5 per group). Schematic representation of the Whole blood thrombolysis assay principle (F). Whole blood thrombolysis assay demonstrated that the activation time (G), the clot 50% lysis (H) and the maximum rate of clot degradation (CLRmax) (I) showed no differences between free r-tPA or r-tPA grafted on the IO@PDA (2-way ANOVA, multiple comparison, n = 5 per group). Data are represented as mean ± SD, *p < 0.05, **p < 0.01, ***p < 0.001, ****p < 0.0001, and ns = not significant, p > 0.05.

**Figure 3:**
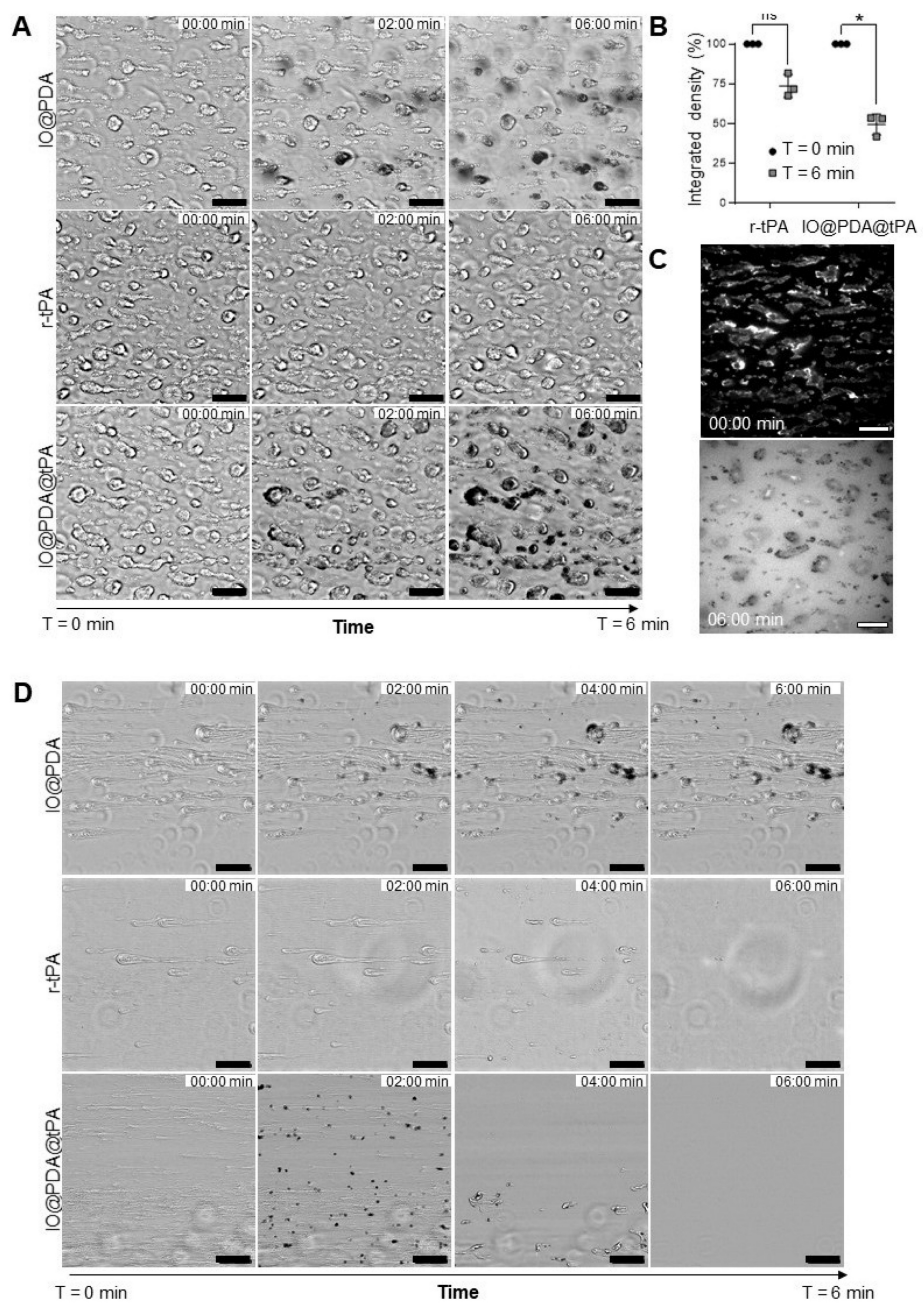
** IO@PDA@tPA effectively target fibrin rich clots *in vitro* while preserving fibrin specific activity.** Representative images of fibrin-rich clots showing IO@PDA and IO@PDA@tPA attachment and lyse versus r-tPA (A). Quantification of integrated density reveals a significant decrease in IO@PDA@tPA-treated clots, while r-tPA shows no significant effect (B). Immunostaining demonstrates complete fibrin fiber lysis by IO@PDA@tPA (C). In the fibrin-only model, IO@PDA particles adhere to fibers without inducing lysis. Both r-tPA and IO@PDA@tPA induce rapid fibrin degradation, starting at 2 min and completing within 6 min (D). Scale bar = 50 µm.

A spectrofluorimetric assay confirmed that r-tPA retained its activity after conjugation to IO@PDA (Figure 2A-B), with activity levels correlating to the respective r-tPA concentration (Figure S2A). Early experiments revealed that using non-dialyzed r-tPA containing large amounts of arginine as an excipient adversely affected its amidolytic activity when grafted onto IO@PDA (Figure S2C-D), probably by interfering with r-tPA binding. Of note, buffer composition of the suspension of IO@PDA@tPA also influenced r-tPA activity, with HEPES buffer negatively affecting its amidolytic activity (Figure S2E-F). This led us to use 0.3 M mannitol in the IO@PDA@tPA suspension. Furthermore, we determined that the r-tPA concentration must be at least twice that of the IO@PDA concentration (in mass) to reach a detectable amidolytic activity of the final particles (Figure S2G-H). To confirm that the optimal IO@PDA@tPA formulation preserved not only the amidolytic but also the fibrinolytic activity, plasma clot lysis assays (platelet-poor clots, Figure 2C) were performed. The results demonstrated that IO@PDA@tPA do not interfere with clot formation (Figure 2D), and that their thrombolytic efficacy was comparable to free r-tPA at the same amidolytic dose (Figure 2E). This was further confirmed by the whole blood halo assay (platelet-rich clots, Figure 2F-G-H-I), which revealed similar clot degradation profiles between IO@PDA@tPA and free r-tPA. Finally, we performed a microfluidic assay to evaluate the specific targeting of IO@PDA@tPA to fibrin-rich clots and its fibrinolytic efficacy on fibrin fibers (Figure 3). In fibrin-rich clots, both IO@PDA and IO@PDA@tPA accumulated around the clot (Figure 3A), with quantification showing a significant increase in IO@PDA@tPA integrated density from the beginning to the end of the acquisition (Figure 3B). Fibrin staining further confirmed that IO@PDA@tPA effectively degraded fibrin within the clots (Figure 3C). In the fibrin fiber microfluidic assay (Figure 3D), IO@PDA attached to fibrin fibers without inducing lysis, whereas IO@PDA@tPA completely degraded the fibers, comparable to r-tPA alone.

### IO@PDA@tPA targets microthrombi in acute IS

**Figure 4:**
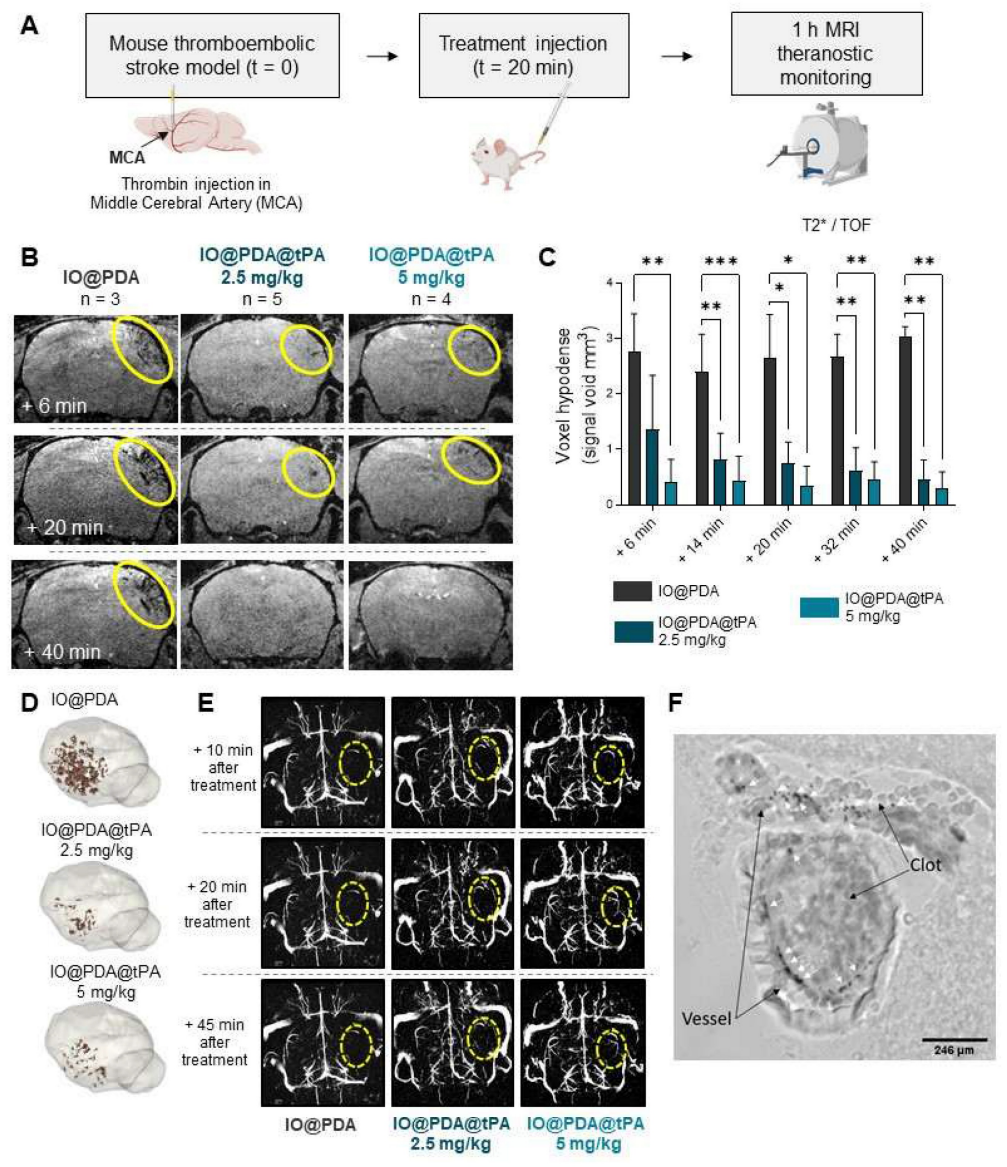
*** Theranostic effects of the IO@PDA@tPA during the acute phase of ischemic stroke.*** Protocol illustration of the intravenous injection of IO@PDA; IO@PDA@tPA 2.5 or 5 mg/kg 20 min after the thromboembolic stroke model. One-hour of MRI acquisition with alternating T_2_^*^; TOF sequences to visualize microthrombi and the main vasculature, respectively, was then performed to monitor thrombolysis (A). Representative T_2_^*^-weighted images of microthrombi depending on the treatment (B). Microthrombi quantification after treatment injection in a mouse model of ischemic stroke (2-way ANOVA, multiple comparisons) (C). 3D representation of microthrombi after IO@PDA; IO@PDA@tPA 2.5 mg/kg and IO@PDA@tPA 5 mg/kg injection (D). Magnetic resonance angiography acquisition to visualize MCA recanalization after treatment injection (E). Brightfield image showing IO@PDA@tPA within clots (F). Data are represented as mean ± SD, *p < 0.05, **p < 0.01, ***p < 0.001, ****p < 0.0001, and ns = not significant, p > 0.05.

Following *in vitro* characterization, we tested their efficacy in a mouse model of IS, induced by thrombin injection in the MCA 17, known to induced microthrombi. Thanks to the magnetic properties of the particles, it is possible to visualize the microthrombi by MRI and to monitor their lysis during the AIS 16. Thus, we first studied the theranostic effects of the IO@PDA@tPA at different doses (Table 1) during the AIS. We injected either IO@PDA (2 mg Fe/kg | 0 mg/kg r-tPA) or IO@PDA@tPA (2 mg Fe/kg | 2.5 mg/kg r-tPA or 4 mg Fe/kg | 5 mg/kg r-tPA) 20 min after inducing the IS in the mice (Figure 4A).

**Figure 5:**
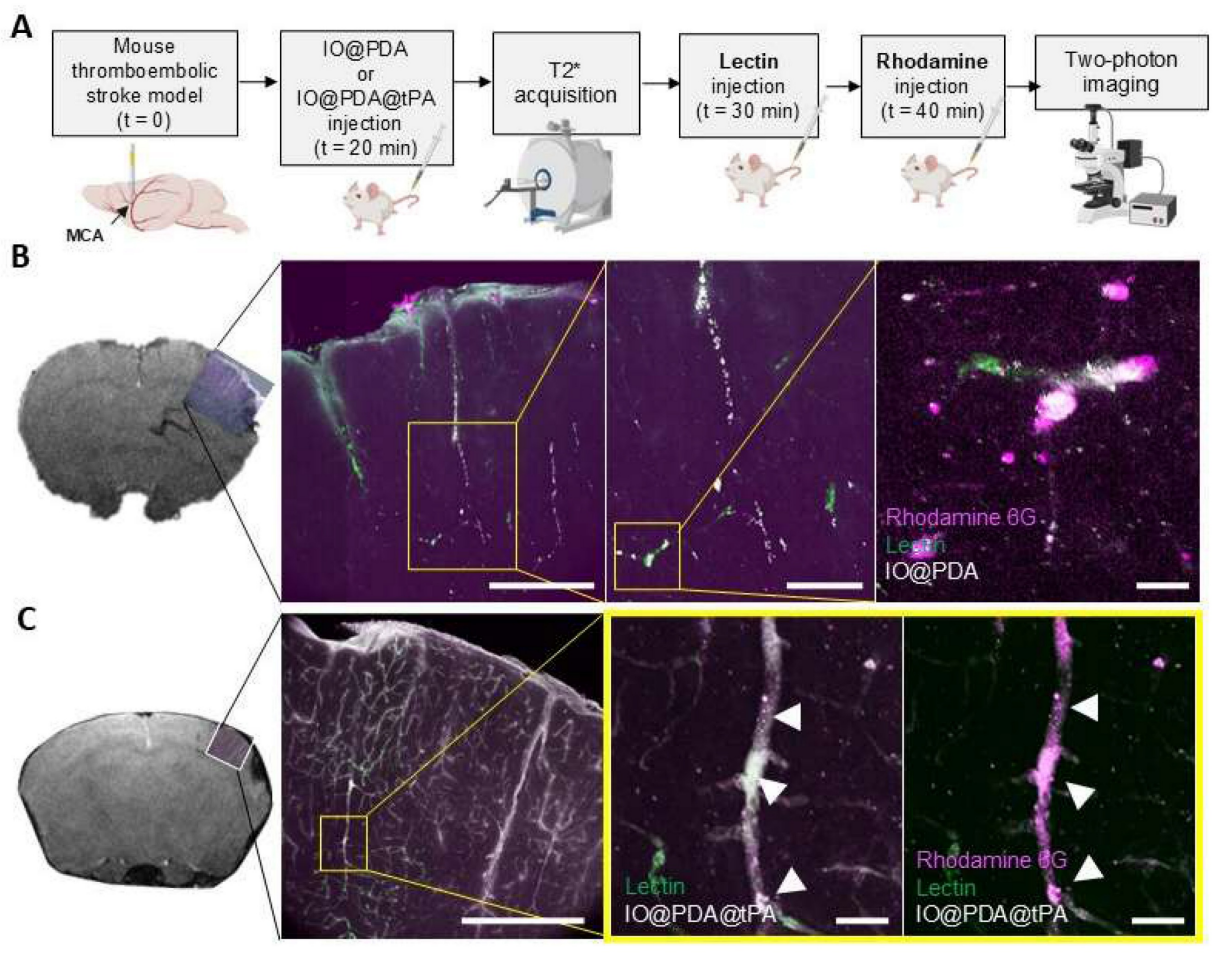
** IO@PDA and IO@PDA@tPA are effectively localized in microthrombi.** Experimental design (A). MRI acquisition and corresponding IHC: IO@PDA in white, microthrombi in magenta (Rhodamine 6G) and vessels in green (lectin) (B). MRI acquisition and corresponding IHC: IO@PDA@tPA in white, microthrombi in magenta (Rhodamine 6G) and vessels in green (lectin) (C). Scale bar = 20 µm.

A one-hour MRI serial acquisition was then performed to visualize microthrombi and early recanalization (Figure 4A, B, E). As expected, we observed a strong hypointense signal on T_2_^*^-weighted images, corresponding to the accumulation of the IO@PDA within microthrombi, after their injection in mice (Figure 4B). In contrast, the hypointense signal visible for the mice treated with the IO@PDA@tPA appears more discrete than the one obtained with the IO@PDA (Figure 4B), as confirmed by the significant decrease of the signal void area (Figure 4C). A 3D representation of the remaining microthrombi further demonstrated the efficacy of IO@PDA@tPA in lysing microthrombi (Figure 4D). This represents a significant advancement over traditional imaging techniques, which often fail to detect microthrombi, leaving them untreated and contributing to further ischemic damage. To ensure that the signal loss was due to the direct lysis of the microthrombi by IO@PDA@tPA and not due to ineffective targeting caused by the grafted r-tPA, we performed an additional experiment: After treating the mice with the IO@PDA@tPA, we injected, right after the one-hour MRI acquisition, the IO@PDA (Figure S4A). As no additional microthrombi signal was observed, we confirmed that the loss of the hypointense signal corresponds to microthrombi lysis (Figure S4B-C). Angiography acquisitions revealed early recanalization in mice treated with IO@PDA@tPA (Figure 4E), foreshadowing a reduced lesion size at 24 h. Immediately after MRI acquisition, brains were collected to assess IO@PDA@tPA localization within clots, which was indeed confirmed (Figure 4F). To strengthen these findings, we performed an experiment in which lectin (for vessel staining) and rhodamine 6G (for clot staining) were injected immediately after IO@PDA or IO@PDA@tPA administration, followed by MRI acquisition and corresponding high-resolution imaging *via* two-photon microscopy (Figure 5A-C). This experiment confirmed that both IO@PDA and IO@PDA@tPA localized within microthrombi (Figure 5B-C).

### IO@PDA@tPA improves outcome in non-diabetic mice

**Figure 6:**
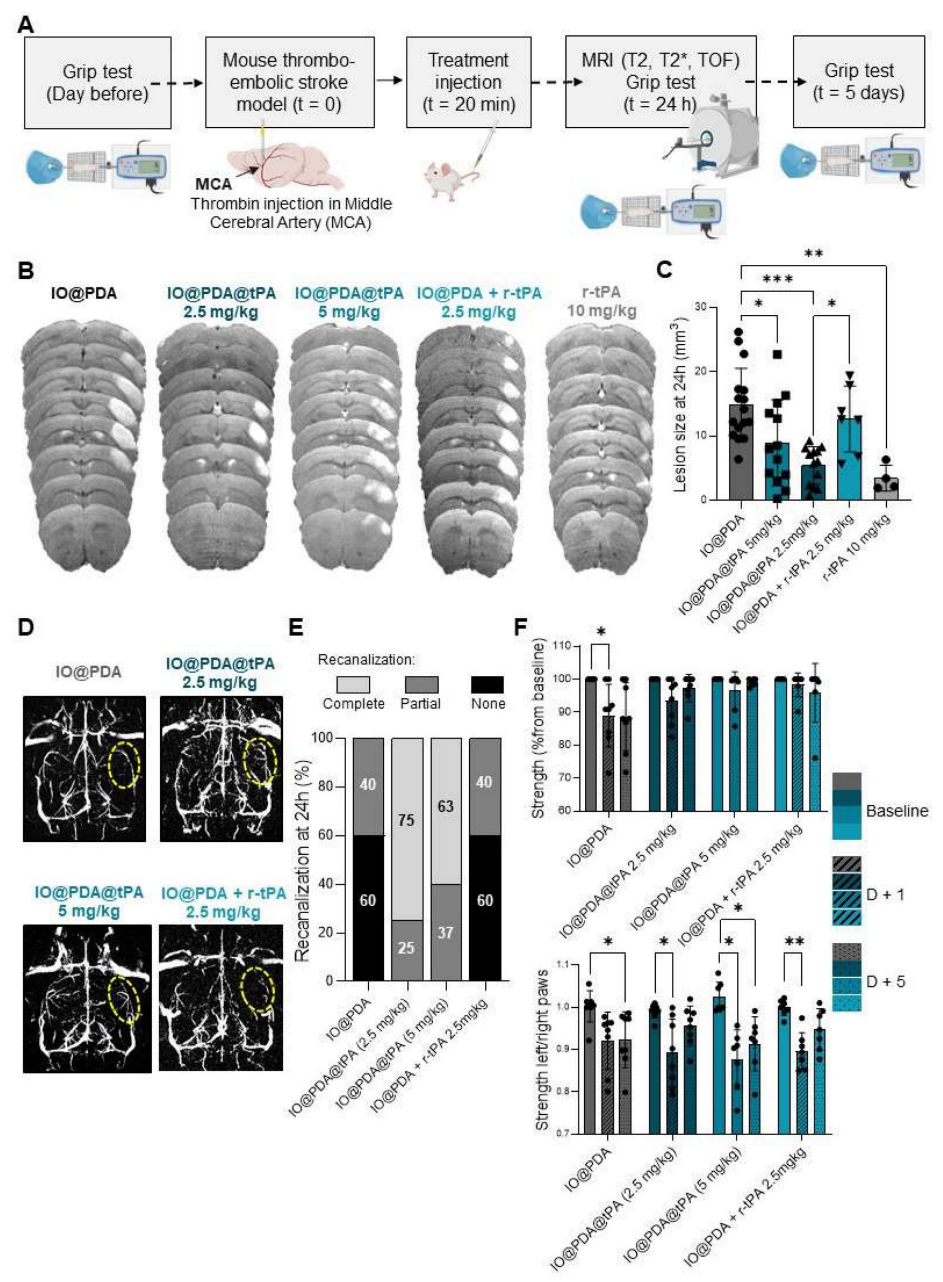
** IO@PDA@tPA 2.5 mg/kg has a beneficial effect on lesion size, recanalization at 24 h after ischemic stroke, and functional recovery.** Protocol illustration of the intravenous injection of IO@PDA; IO@PDA@tPA 2.5 or 5 mg/kg; IO@PDA + r-tPA 2.5 mg/kg or r-tPA 10 mg/kg, 20 min after the thromboembolic stroke model. *T_2_*-weighted images for the lesion size, *T_2_s*-weighted images for hemorrhagic transformation and TOF sequences for the vasculature were acquired 24 h after IS by MRI. Finally, to assess functional recovery, grip test was performed the day before inducing the ischemic stroke and at day 1 and day 5 after stroke onset (A). Lesion size at 24 h after injection of IO@PDA; IO@PDA@tPA 2.5 mg/kg; IO@PDA@tPA 5 mg/kg; IO@PDA + r-tPA 2.5 mg/kg or r-tPA 10 mg/kg infusion (B). IO@PDA@tPA 2.5 mg/kg injection significantly decreased the lesion volume compared to the other groups and show no differences with the r-tPA 10 mg/kg infusion treated mice (One-way ANOVA, multiple comparisons, n = 4-16) (C). Representative angiographies of IO@PDA; IO@PDA@tPA 2.5 mg/kg; IO@PDA@tPA 5 mg/kg; IO@PDA + r-tPA 2.5 mg/kg treated groups 24 h after stroke onset (D). Recanalization score 24 h post IS depending of the treatment administered (E). Functional recovery: evaluation of the global strength of the forepaw (upper row) and the specific left paw strength deficit measured by the grip-test ratio (strength of the left paw relative to the right paw) (bottom row; 2-way ANOVA, multiple comparisons, n = 5-10) (F). Data are represented as mean ± SD, *p < 0.05, **p < 0.01, ***p < 0.001, ****p < 0.0001, and ns = not significant, p > 0.05.

We then assessed the potential effect of the IO@PDA@tPA on lesion size and recanalization of the main occluded artery 24 h after IS, and on the functional recovery at 1 day and 5 days after stroke (Figure 6A). Mice treated with the IO@PDA@tPA at 5 mg/kg and 2.5 mg/kg showed a significant decrease of the lesion size at 24 h compared to mice treated with the IO@PDA or the classical r-tPA treatment (10% bolus, 90% perfusion, Figure 6B-C). To confirm that this result was due to specific targeting of the IO@PDA@tPA to thrombi and not the effect of r-tPA alone, we added another group as a mechanistic control in which IO@PDA was injected first, followed by r-tPA at 2.5 mg/kg. Mice treated with IO@PDA + r-tPA at 2.5 mg/kg had significantly larger lesions than those treated with IO@PDA@tPA 2.5 mg/kg (Figure 6B-C). Additionally, no significant difference in lesion size was observed between mice treated with IO@PDA or IO@PDA + r-tPA 2.5 mg/kg (Figure 6B-C).

Angiographies revealed a majority of animals with no recanalization in the IO@PDA and IO@PDA + r-tPA 2.5 mg/kg condition but 75% and 68% of complete recanalization at 24 h after IO@PDA@tPA 2.5 mg/kg and 5 mg/kg administration respectively (Figure 6D-E). Thus, mice treated with IO@PDA@tPA required a r-tPA dose four times lower than the standard thrombolytic therapy with free r-tPA (10 mg/kg), demonstrating enhanced therapeutic efficacy. This implies that IO@PDA@tPA allows a better recanalization rate but also enhances reperfusion in the penumbra. This finding is crucial, as reperfusion in the penumbra is often insufficient with conventional therapies, leading to extended ischemic damage and worsen outcomes 2-5. However, the variability in lesion size among mice treated with IO@PDA@tPA at 5 mg/kg suggests the possibility of a U-shaped dose-response curve. Thus, we have then tested lower doses of IO@PDA@tPA (0.5 and 1 mg/kg) and included a saline control group (Figure S4D). At 0.5 mg/kg, IO@PDA@tPA produced a reduction in lesion size that was not significantly different from IO@PDA or saline conditions, thereby supporting the U-shaped dose-response relationship and confirming 2.5 mg/kg as the optimal dose for IS treatment. The saline group showed no significant reduction in lesion size and displayed greater interindividual variability, indicating a lack of spontaneous recovery and reinforcing the therapeutic relevance of IO@PDA@tPA even at low doses. Interestingly, the IO@PDA-only group (without r-tPA) exhibited a trend toward lesion size stabilization compared to saline, possibly due to clot-stabilizing effects, consistent with our previous study 16. Finally, mice treated with IO@PDA@tPA exhibited a full functional recovery at 5 days after stroke whereas the mice treated with IO@PDA or IO@PDA + r-tPA 2.5 mg/kg still had strength deficit of the left paw (Figure 6F). Moreover, at 24 h post-IS, no correlation was observed between lesion size and functional recovery (Figure S5A, C, E, G). In contrast, at 5 days post-IS, mice treated with IO@PDA or IO@PDA@tPA (2.5 and 5 mg/kg) showed a negative correlation between lesion size and functional recovery, indicating that larger lesions were associated with poorer recovery (Figure S5B, D, F, H).

**Figure 7:**
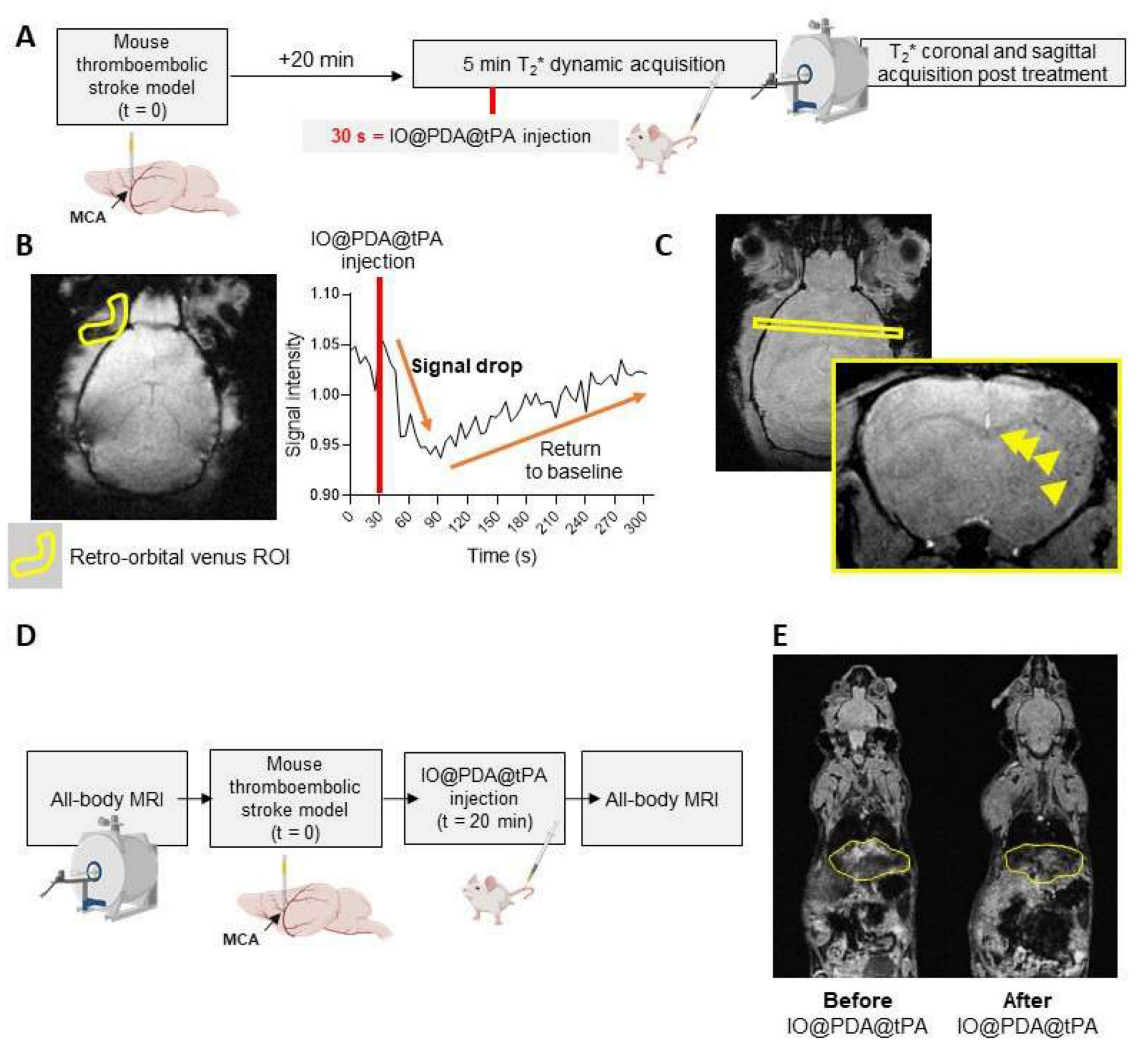
** Pharmacokinetics and biodistribution of IO@PDA@tPA.** Experimental design (A). Representative T_2_^*^ images obtained during the 5-min dynamic acquisition with ROI placement (left) and corresponding signal intensity curve (right), showing a sharp signal drop in the retro-orbital venous ROI after IO@PDA@tPA injection, followed by a return to baseline within 30 s (B). T_2_^*^ acquisition showing IO@PDA@tPA bound to residual microthrombi (C). Protocol design for the IO@PDA@tPA biodistribution study (D). Protocol and representative whole-body MRI images before and after IO@PDA@tPA injection.

We also studied the pharmacokinetics and biodistribution of the IO@PDA@tPA (Figure 7). To do so, we performed a 5-minute T_2_^*^ dynamic acquisition to measure the signal drop in the retro-orbital venous of the mouse after the IO@PDA@tPA injection (Figure 7A, B). We also performed a T_2_^*^ coronal and sagittal acquisition post-treatment to confirm the presence of microthrombi (Figure 7A, C). To estimate the half-life, we set the injection time to t_inj_ =30 s (Figure 7B). The injection was performed at t_inj_ = 30 s, and only data points with t ≥ 30 s were considered as post-injection values (the first point available was at 30.72 s). Relative time was defined as t′ = t - t_inj_, such that t′ = 0 corresponds to the time of injection (Figure 7B). To account for the signal decrease following injection, the deviation from baseline was calculated as C(t) = B - Y(t), where B represents the mean pre-injection signal (Figure 7B). A semi-logarithmic fit of lnC(t) versus t′ yielded an elimination rate constant of k ≈ 3.435×10^-3^ s^-1^, corresponding to a half-life of t_1/2_ ≈ 201.8 s. Although the first post-injection measurement was obtained at t′ = 0.72 s rather than exactly at t′ = 0, this discrepancy had a negligible impact on the estimation of k. Finally, since r-tPA is conjugated to IO@PDA, the biodistribution of IO@PDA@tPA is expected to follow that of free IO@PDA previously reported 18. In that study, we showed that the MRI signal returned to baseline levels within 7 days in the liver and within one month in the spleen, with no significant accumulation or signal change in the kidneys of mice. To confirm the biodistribution profile of IO@PDA@tPA, we performed whole-body MRI (Figure 7D), which revealed a signal drop in the liver (Figure 7E), thereby confirming a biodistribution pattern consistent with our previous findings.

### Opposing immune effects of polydopamine and IO@PDA@tPA in IS

**Figure 8:**
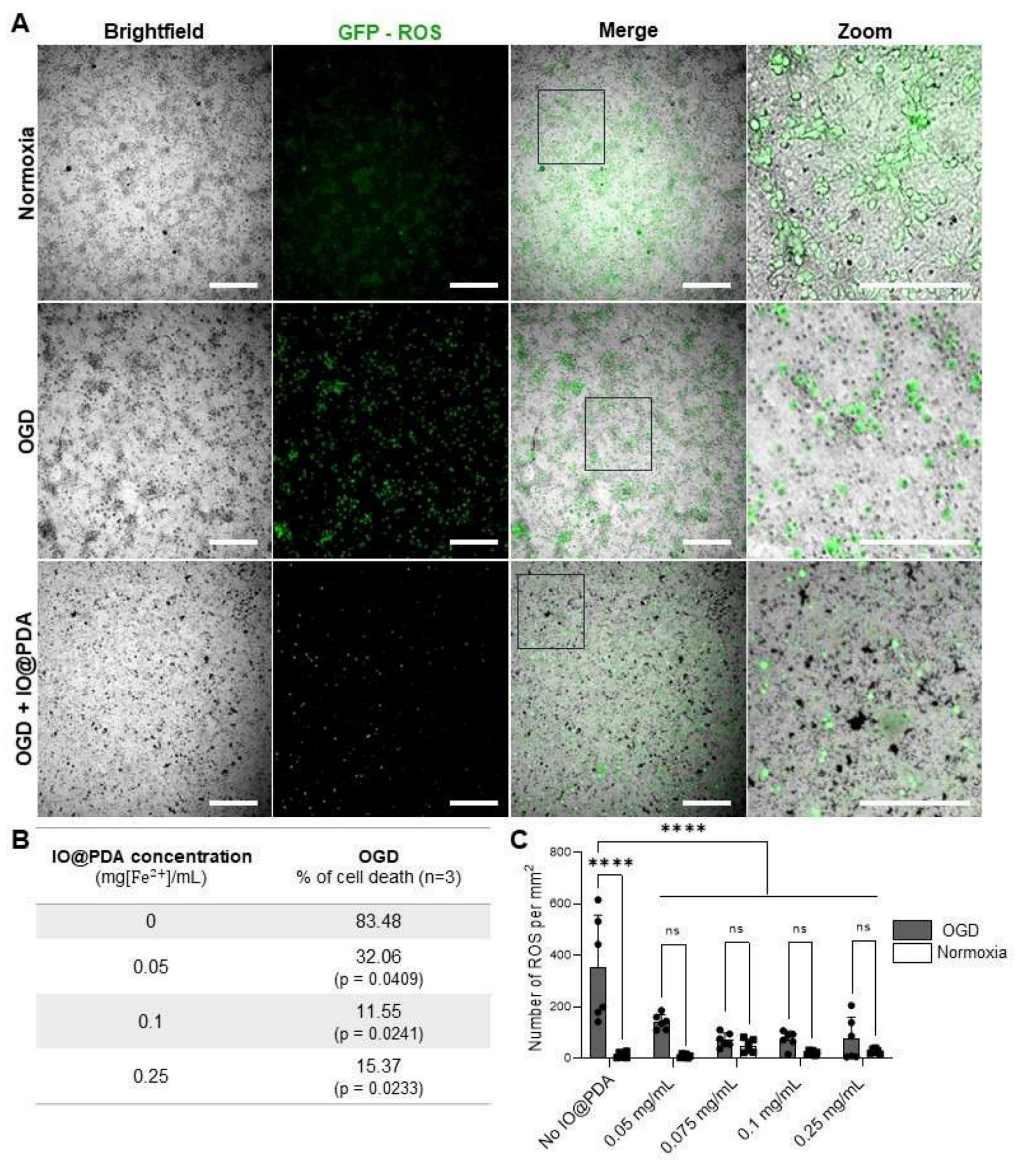
** IO@PDA decrease Reactive Oxygen Species (ROS) secretion and rescue neurons from death under Oxygen Glucose Deprivation (OGD) condition.** Illustrations of ROS (in green) in OGD versus normoxy conditions in the absence or presence of IO@PDA (in black) (A). IO@PDA was able to reduce neuronal cell death under OGD conditions (2-way ANOVA, multiple comparisons, n = 3) (B). ROS secretion was significantly decreased by IO@PDA compared to control conditions (2-way ANOVA, multiple comparisons, n = 6) (C). Scale bar = 312 µm.

Given the antioxidant properties of polydopamine, we first assessed whether IO@PDA exposure could have deleterious effects on neuronal viability *in vitro*. To this end, we used primary neuronal cultures subjected to oxygen and glucose deprivation (OGD) to mimic ischemic conditions. Our results demonstrated that IO@PDA was not neurotoxic; on the contrary, their presence reduced neuronal cell death and ROS levels under OGD conditions (Figures 8A-C). This experiment therefore served to confirm the safety of IO@PDA particles for neuronal survival under stress conditions.

**Figure 9:**
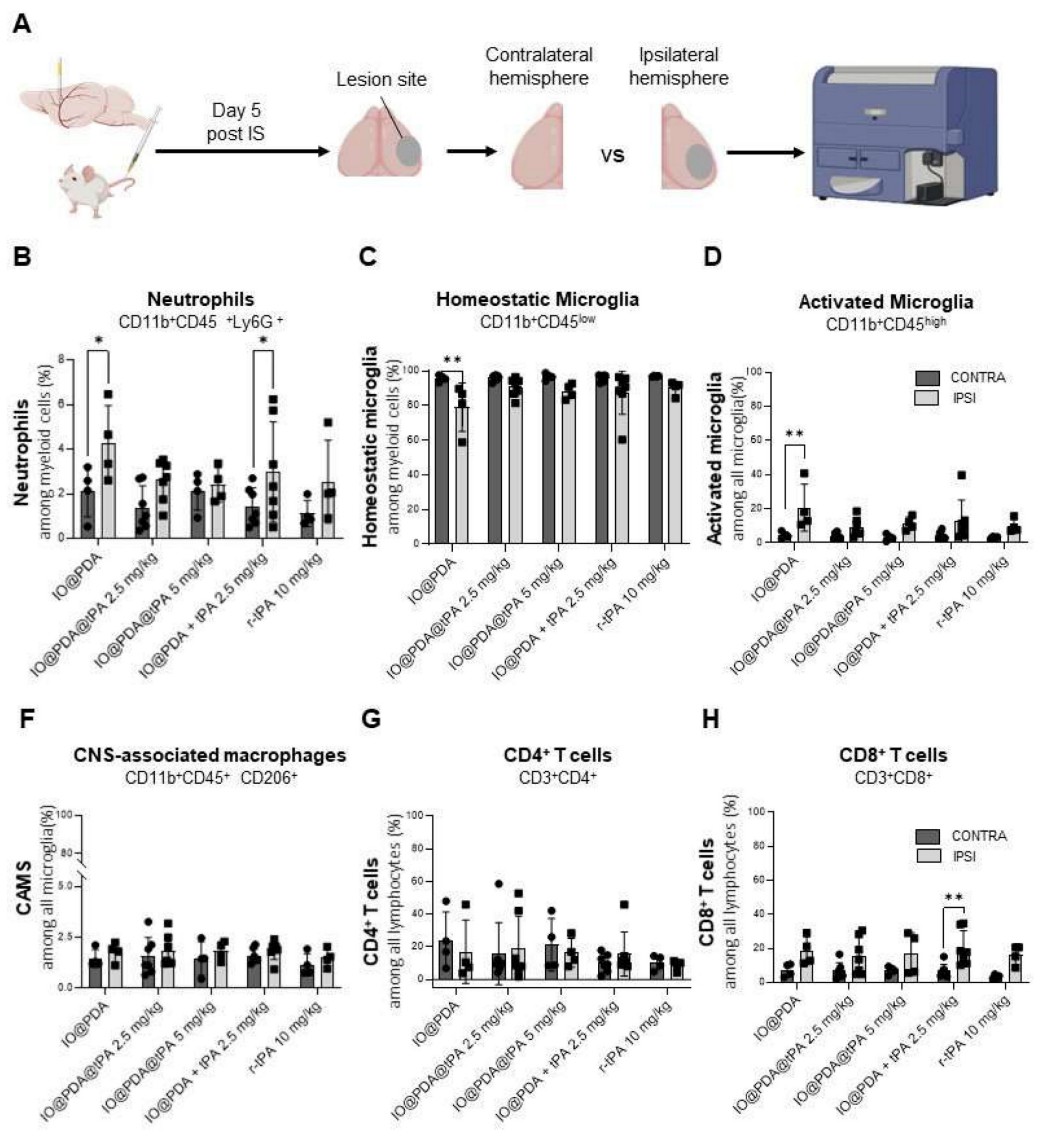
** IO@PDA increases neutrophil infiltration and microglial activation *in vivo*.** Scheme of the flow cytometry protocol for the *ex vivo* analysis of immune cell infiltrates in the brains. Gating strategy is explicated in figure S4 (A). Quantification of neutrophil frequency (%) among myeloid cells in the contralateral (control) and ipsilateral (lesion site) hemispheres (2-way ANOVA, multiple comparisons, n = 4-7) (B). Quantification of homeostatic microglia frequency (%) among myeloid cells in the contralateral and ipsilateral hemispheres (2-way ANOVA, multiple comparisons, n = 4-7) (C). Quantification of activated microglia (% among total microglia) in the contralateral and ipsilateral hemispheres (D). Quantification of CAMS (% among total microglia) in the contralateral and ipsilateral hemispheres (2-way ANOVA, multiple comparisons, n = 4-7) (E). Quantification of CD4⁺ T cells frequency (%) in the contralateral and ipsilateral hemispheres (F). Quantification of CD8⁺ T cells (% among total lymphocytes) in the contralateral and ipsilateral hemispheres (2-way ANOVA, multiple comparisons, n = 4-7) (G). Data are represented as mean ± SD, *p < 0.05, **p < 0.01, ***p < 0.001, ****p < 0.0001, and ns = not significant, p > 0.05.

To further investigate potential *in vivo* effects, we analyzed inflammatory processes 5 days post-IS by flow cytometry, comparing the ipsilateral hemisphere (lesion site) to the contralateral hemisphere (control) (Figure 9A). We focused on myeloid populations—specifically neutrophils, activated microglia, and CNS-associated macrophages (CAMs) (Figure S6A)—as well as on T cell populations (Figure S6B). Results showed a significant increase in neutrophil infiltration in the ipsilateral hemisphere of mice treated with IO@PDA and IO@PDA + r-tPA 2.5 mg/kg (Figure 9B) compared to the contralateral side. In contrast, mice treated with IO@PDA@tPA (2.5 and 5 mg/kg) or r-tPA alone (10 mg/kg) showed no significant difference in neutrophil levels between hemispheres (Figure 9B). Interestingly, mice treated with IO@PDA alone exhibited a significant reduction in homeostatic microglia in the ipsilateral cortex (Figure 9C), along with a significant increase in activated microglia (Figure 9D). In contrast, mice treated with IO@PDA@tPA (2.5 and 5 mg/kg), IO@PDA + r-tPA 2.5 mg/kg, or r-tPA 10 mg/kg did not show significant differences in the number of homeostatic or activated microglia between hemispheres (Figures 9CD). For CAMs, no significant hemispheric differences were observed across all treatment groups (Figure 9E). Similarly, the number of CD4+ T cells remained unchanged between hemispheres (Figure 9F). However, there was a trend toward increased CD8+ T cells in the ipsilateral hemisphere across all groups, reaching statistical significance in IO@PDA + r-tPA 2.5 mg/kg-treated mice (Figure 9G). Altogether, these data demonstrate that while IO@PDA particles are not neurotoxic and even promote neuronal survival under *in vitro* ischemic stress, they elicit a pro-inflammatory response *in vivo*, characterized by neutrophil infiltration and microglial activation. Importantly, these immunostimulatory effects were absent when r-tPA was conjugated to the particles (IO@PDA@tPA), as immune cell profiles remained comparable between contralateral and ipsilateral hemispheres.

### IO@PDA@tPA induces no HT and boosts recovery in diabetes

**Figure 10:**
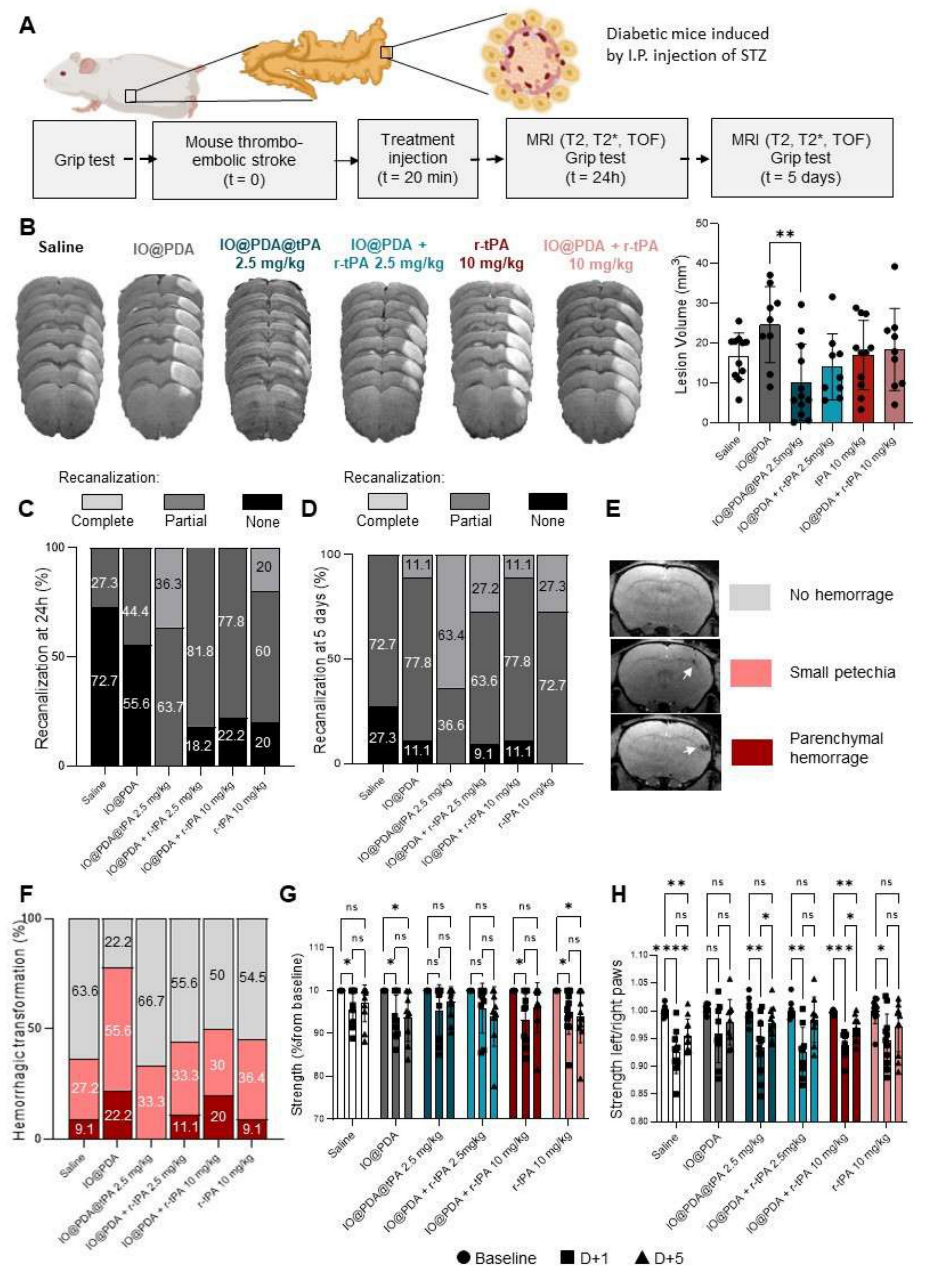
** Theranostic treatment of microthrombi in comorbidity diabetic mice.** Protocol illustration of the intravenous injection of Saline; IO@PDA; IO@PDA@tPA 2.5 mg/kg; IO@PDA + r-tPA 2.5 mg/kg; r-tPA 10 mg/kg; IO@PDA + r-tPA 10 mg/kg 20 min after the thromboembolic stroke model. *T_2_*-weighted images for the lesion size, *T_2_s*-weighted images for hemorrhagic transformation and TOF sequences for the vasculature were acquired 24 h and 5 days after stroke onset by MRI. Finally, to assess functional recovery, grip test was performed the day before inducing the ischemic stroke and at day 1 and day 5 after stroke (A). Lesion size 24 h after injection of Saline; IO@PDA; IO@PDA@tPA 2.5 mg/kg; IO@PDA + r-tPA 2.5 mg/kg; r-tPA 10 mg/kg; IO@PDA + r-tPA 10 mg/kg (One-way ANOVA, multiple comparisons, n = 9-12) (B). Recanalization score at 24 h after stroke (C). Recanalization score at 5 days after stroke. (D). Representation of the hemorrhagic transformation scale for the analysis (E). Hemorrhagic score at 5 days after stoke (F). Quantification of the global strength deficit measured by a grip-test of the forepaws of the mice treated with Saline; IO@PDA; IO@PDA@tPA 2.5 mg/kg; IO@PDA + r-tPA 2.5 mg/kg; r-tPA 10 mg/kg; IO@PDA + r-tPA 10 mg/kg before IS and at 1 and 5 days after IS stroke (2way ANOVA, multiple comparisons, n = 9-12 per group) (G). Quantification of the specific left paw strength deficit measured by a grip-test ratio (strength of the left paw relative to the right paw) of the mice treated with Saline; IO@PDA; IO@PDA@tPA 2.5 mg/kg; IO@PDA + r-tPA 2.5 mg/kg; tPA 10 mg/kg; IO@PDA + r-tPA 10 mg/kg before IS and at 1 and 5 days after IS stroke (2way ANOVA, multiple comparisons, n = 9-12 per group) (H). Data are represented as mean ± SD, *p < 0.05, **p < 0.01, ***p < 0.001, ****p < 0.0001, and ns = not significant, p > 0.05.

Hyperglycemia is a highly prevalent comorbidity among stroke patients. Diabetes increases the risk of stroke and worsens its outcome 18. Thus, we studied the IO@PDA@tPA (2.5 mg/kg) in hyperglycemic mice after stroke induction, in which r-tPA treatment is known to induce HT, to evaluate a potential benefit of IO@PDA@tPA over r-tPA on HT and on its limited efficacy on lesion size 19. To induce hyperglycemia, mice were treated for five days with intraperitoneal injections of STZ (40 mg/kg). After inducing IS, lesion size, recanalization, HT, and functional recovery were assessed, depending on the treatment received (Saline, IO@PDA, IO@PDA@tPA 2.5 mg/kg, IO@PDA + r-tPA 2.5 mg/kg, r-tPA 10 mg/kg, or IO@PDA + r-tPA 10 mg/kg; Figure 10A). These experiments revealed that the mean lesion size at 24 h is significantly decreased in the IO@PDA@tPA 2.5 mg/kg group compared to the IO@PDA-treated mice. Other groups showed reduced but not significant lesion size (Figure 10B).

Angiographies revealed that most mice in the Saline and IO@PDA groups showed no recanalization, while 18%, 22%, and 20% of no recanalization events were observed for the IO@PDA + r-tPA 2.5 mg/kg; IO@PDA + r-tPA 10 mg/kg and r-tPA 10 mg/kg groups, respectively, at 24 h after stroke (Figure 10C). In contrast, IO@PDA@tPA 2.5 mg/kg treated mice showed 36% of complete recanalization at 24 h after stroke onset (Figure 10C). At 5 days after stroke, 63% of the IO@PDA@tPA 2.5 mg/kg treated mice had a complete recanalization and 37% showed partial recanalization (Figure 10D). Mice treated with r-tPA 10 mg/kg showed only 27% of complete recanalization and 73% of partial recanalization. For the other groups we observed the same percentage of partial recanalization as in the mice treated with r-tPA 10 mg/kg but 9 to 27% of them didn't show any recanalization (Figure 10D). *T_2_s*-weighted MRI acquisition revealed no HT 24 h after stroke (Figure S7). However, hypointense signals corresponding to microthrombi were observed in the IO@PDA and the IO@PDA + r-tPA 2.5 mg/kg treated mice confirming that without r-tPA or if r-tPA is not linked to IO@PDA at a lower dose, microthrombi are not completely lysed (Figure S7). At 5 days after stroke, IO@PDA@tPA 2.5 mg/kg treated mice showed no HT (67%) and 33% of small petechiae whereas, 9 to 22% of the mice treated with the other groups exhibited HT, even in the saline treated-group (Figure 10F).

We further investigated the functional recovery by evaluating the global strength of the forepaw and the specific strength of the left paw with a grip test as well as sensorimotor function by a custom-made corridor task, based on the ability of animals to explore objects (Figure 10G-H and Figure S7). The grip test results revealed that the IO@PDA and r-tPA 10 mg/kg treated mice have a global strength deficit at 1 day and 5 days after stroke (Figure 10G). The Saline, IO@PDA + r-tPA 10 mg/kg treated mice had a global strength deficit at 1 day after stroke (Figure 10G); and the IO@PDA@tPA 2.5 mg/kg and IO@PDA + r-tPA 2.5 mg/kg treated mice had no global strength deficit at 1 and 5 days after stroke (Figure 10G).

Regarding left paw strength, IO@PDA-treated mice showed a persistent deficit at 1 and 5 days post-stroke, although this did not reach statistical significance (Figure 6I-H). Mice treated with Saline and IO@PDA + r-tPA (10 mg/kg) exhibited a deficit at both 1 and 5 days but began to recover thereafter (Figure 6I-H). Similarly, r-tPA (10 mg/kg) and IO@PDA@tPA (2.5 mg/kg) as well as IO@PDA + r-tPA (2.5 mg/kg) groups displayed a deficit at 1 day but no measurable deficit at 5 days post-stroke (Figure 6I-H). Interestingly, only IO@PDA@tPA-treated mice exhibited a negative correlation between lesion size and functional recovery at 24 h post-IS (Figure S8A, C, E, G, I, K), which disappeared by 5 days (Figure S8F). In contrast, negative correlations at 5 days post-IS were observed exclusively in mice treated with IO@PDA + r-tPA (10 mg/kg) and r-tPA (10 mg/kg) (Figure S8B, D, F, H, J, L). These findings suggest that IO@PDA@tPA not only promotes early functional recovery independent of lesion size but may also mitigate the long-term impact of lesion burden, whereas high-dose r-tPA regimens remain more tightly linked to lesion volume in determining functional outcome. For the sensorimotor function with the corridor test, all groups displayed a deficit on the number of visited objects at 1 day after stroke (Figure S9). The Saline; IO@PDA; IO@PDA + r-tPA 10 mg/kg; r-tPA 10 mg/kg treated mice still had a deficit on the number of visited objects at 5 days after stroke (Figure S9). The IO@PDA + r-tPA 2.5 mg/kg and the IO@PDA@tPA 2.5mg/kg treated mice no longer had a deficit at 5 days after stroke (Figure S9).

Our findings highlight several advantages of IO@PDA@tPA compared to free r-tPA. At 2.5 mg/kg, IO@PDA@tPA achieved similar lesion size reduction and vascular recanalization as 10 mg/kg of free r-tPA, representing a fourfold dose reduction that could lower the risk of systemic hemorrhagic complications. The fibrinogen-enriched protein corona enabled thrombus-specific accumulation, as confirmed by MRI, immunohistochemistry, and microfluidic assays, whereas free r-tPA lacks targeting capacity and acts systemically. IO@PDA@tPA treatment was also associated with balanced immune profiles, in contrast to IO@PDA alone, and IO@PDA particles did not increase ROS production in primary neurons under OGD, indicating neuronal compatibility. Despite these advantages, several limitations remain. Variability in lesion size and functional outcome was observed across treatment groups, both in non-diabetic and diabetic cohorts. This variability likely reflects intrinsic heterogeneity of the thrombin MCAO model, including differences in occlusion severity, collateral circulation, and reperfusion dynamics, which are further amplified in diabetic mice due to vascular dysfunction and inflammatory priming. Moreover, while IO@PDA@tPA at 5 mg/kg was safe and effective, the absence of a strict dose-response relationship suggests biological saturation. Once fibrin availability or local enzymatic activity at the clot site is maximally engaged, increasing the dose may not provide further benefit, and could instead contribute to variability through local dispersion or off-target interactions, even though we did not observe hemorrhagic transformation or toxicity at the highest tested dose. In addition, as suggested by our immune profiling, subtle immunomodulatory effects at higher doses could also influence outcomes, particularly in diabetic mice where immune dysregulation is prominent. IO@PDA@tPA is a complex multifunctional nanoconjugate requiring rigorous physicochemical characterization, reproducibility, and scalability before clinical translation, unlike free r-tPA which is already approved and widely available. Furthermore, IO@PDA clearance from liver and spleen is slower than the systemic clearance of free r-tPA, which may raise regulatory considerations, although no toxicity was observed in our models. Importantly, both PDA and IO are known to be biodegradable: PDA undergoes slow oxidative degradation through macrophage processing, while IO nanoparticles are metabolized *via* endogenous iron pathways. In line with our previous work 16, 20, we observed transient liver and spleen accumulation without toxicity, further supporting the favorable biocompatibility of IO@PDA@tPA. Finally, while the benefits of IO@PDA@tPA are well documented, the precise mechanisms underlying immune modulation and thrombus targeting require further investigation, including studies in larger animal models and in the context of hemorrhagic risk.

## Conclusions

In this study, we present a microthrombi theranostic agent IO@PDA@tPA that combines promising characteristics to improve thrombolysis therapy in acute ischemic stroke. The vectorization of the r-tPA to the edge of the thrombi combined with the antioxidant property resulted in (i) an improved beneficial effect in a non-diabetic and a diabetic group, (ii) at a four times lower dose than usually used in clinic, (iii) an improvement of functional score as measured by the behavior testing, (iv) without inducing hemorrhagic transformation. The dual action of IO@PDA@tPA in detecting and degrading microthrombi offers a significant advancement over current stroke therapies, particularly for patients with comorbidities like diabetes, where conventional treatments may fail. Future research will focus on validating these findings in clinical settings and exploring the broader applicability of this theranostic approach to other thrombotic conditions.

## Supplementary Material

Supplementary figures.

## Figures and Tables

**Table 1:** Summary of the particle's properties

	IO@PDA	IO@PDA@tPA	
Hydrodynamic diameter (nm) ± SD	614.6 ± 74.5	615.2 ± 80.3	
Zeta potential (mV) ± SD	-23.79 ± 3.6	-22,64 ± 2.9	
Iron doses injected to mice (mg[ 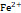 ]/kg)	2	2	4
r-tPA doses injected to mice (mg/kg)	0	2.5	5

## Data Availability

All data generated or analyzed in this study are included in this published article and its supplementary information files.

## Abbreviations

A_t_: activation time
AIS: acute ischemic stroke
BSA: bovine serum albumin
CL: clotting time
CLRmax: maximum rate of clot degradation
DCFDA: Dichlorodihydrofluorescein Diacetate solution
DIV: days *in vitro*
D_max_: maximum degradation
DMEM: Dulbecco's Modified Eagle medium
DWI: Diffusion-weighted images
FLASH: fast-low angle shot
HT: hemorrhagic transformation
IO: iron oxide
IO@PDA: iron oxide microparticles surrounded by polydopamine
IO@PDA@tPA: iron oxide microparticles surrounded by polydopamine conjugated with r-tPA
I.P.: intraperitoneal
IS: ischemic stroke
LDH: lactate dehydrogenase
LT: lysis time
MCA: middle cerebral artery
MRA: magnetic resonance angiography
MRI: magnetic resonance imaging
OGD: oxygen and glucose deprivation
PBS: phosphate buffer saline
PDA: polydopamine
PHySIOMIC: Polydopamine Hybridized Superparamagnetic Iron Oxide Mussel Inspired Clusters
RARE: Rapid Acquisition with Relaxation
ROS: reactive oxygen species
r-tPA: recombinant tissue-type plasminogen activator
SD: standard deviation
STZ: streptozotocin
TOF: time of flight
r-tPA: recombinant tissue-type plasminogen activator
