# Supplementary material for: Engineered nanoconjugates for simultaneous detection and degradation of stroke-associated microthrombi
Source: Theranostics. 2026 Jan 1;16(3):1082–102. doi: 10.7150/thno.119705 (PMC12679082; doi:10.7150/thno.119705)
Supplement: Supplementary file 1 — Supplementary figures. [file thnov16p1082s1.pdf]

Supplementary Data

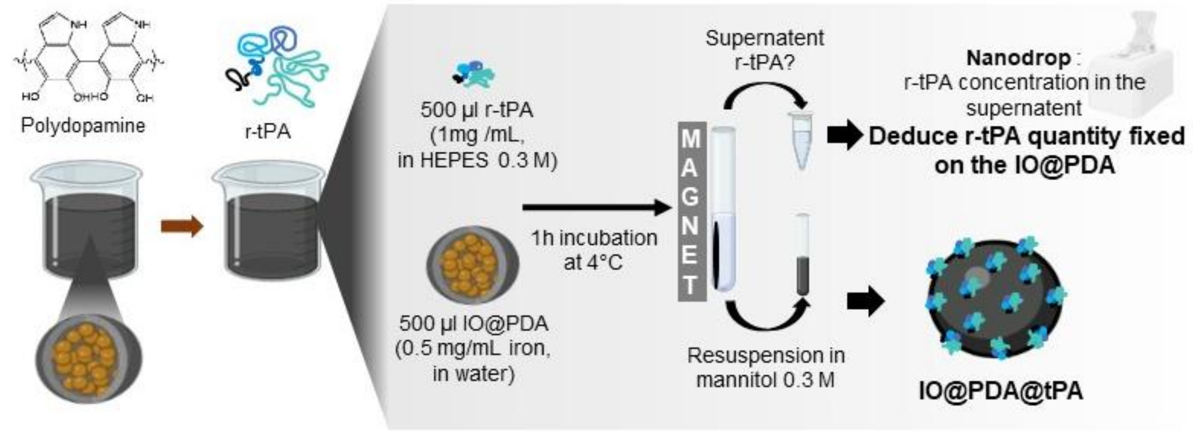

Supplementary Figure 1. Protocol illustration of IO@PDA functionalization with r-tPA.

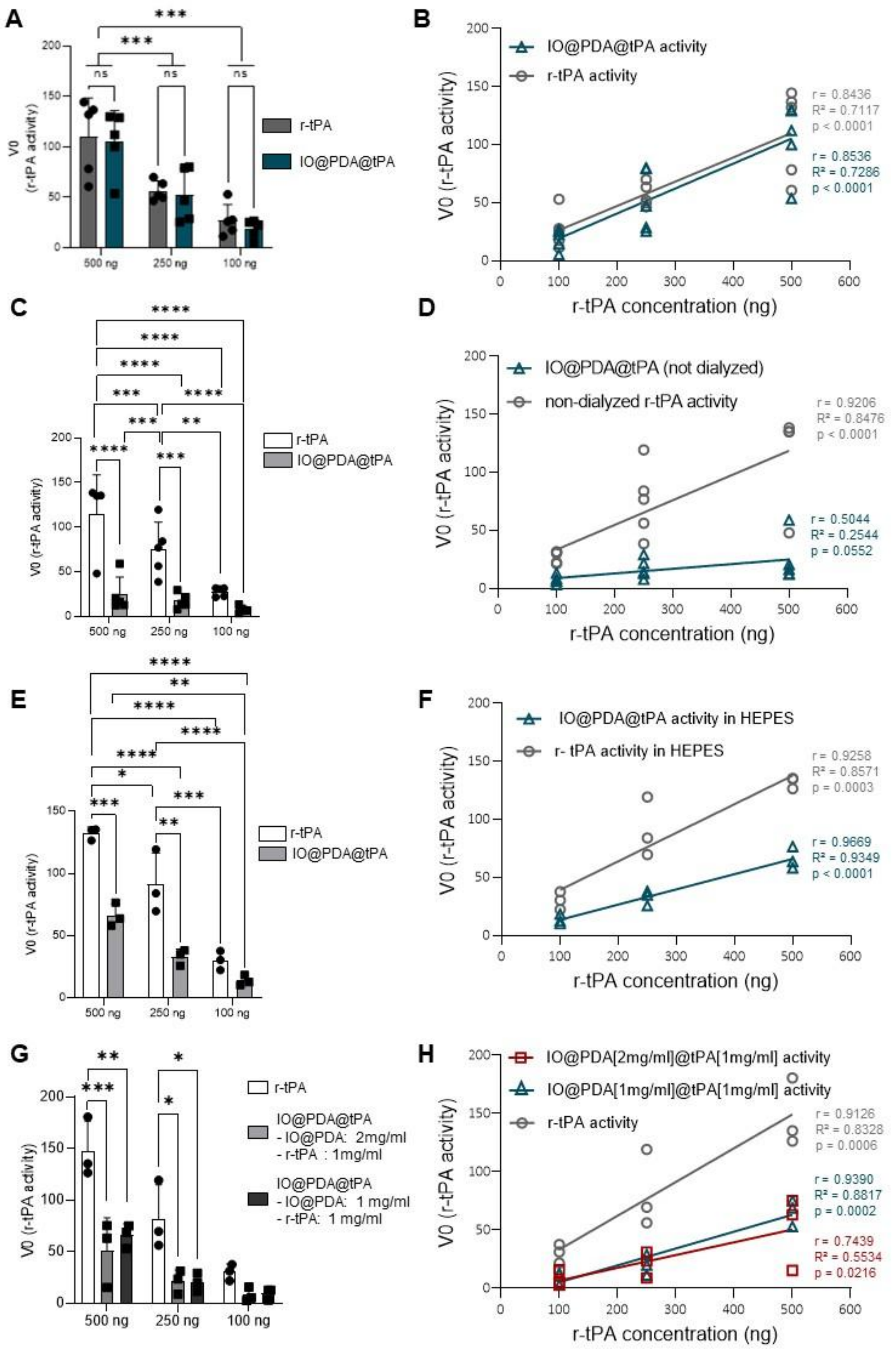

Supplementary Figure 2. Optimization of the r-tPA Coupling Method on Particles. Final IO@PDA@tPA optimization with dialyzed r-tPA with IO@PDA@tPA resuspension in Mannitol (0.3M) demonstrated no loss of r-tPA activity (A) and a strong correlation between the amount of r-tPA and its activity. There was no difference between the two-correlation slope reinforcing the precedent results (B). First studies using non-dialyzed r-tPA showed a loss of r-tPA activity when grafted to the IO@PDA particles (C). The correlation slope strongly supported a loss of amidolytic activity of non-dialyzed r-tPA when conjugated on the IO@PDA (D). Other attempts with dialyzed r-tPA with IO@PDA@tPA resuspension in HEPES (0.3M) instead of mannitol (0.3M) again revealed a loss of r-tPA activity when coupled to the IO@PDA (E, F). Finally, increasing the concentration of IO@PDA 2-fold higher than the concentration of r-tPA showed once again a loss of r-tPA activity when linked on the particles (G, H). Data are represented as mean ± SD, $*p < 0.05$, $**p < 0.01$, $***p < 0.001$, $****p < 0.0001$, and ns = not significant, $p > 0.05$.

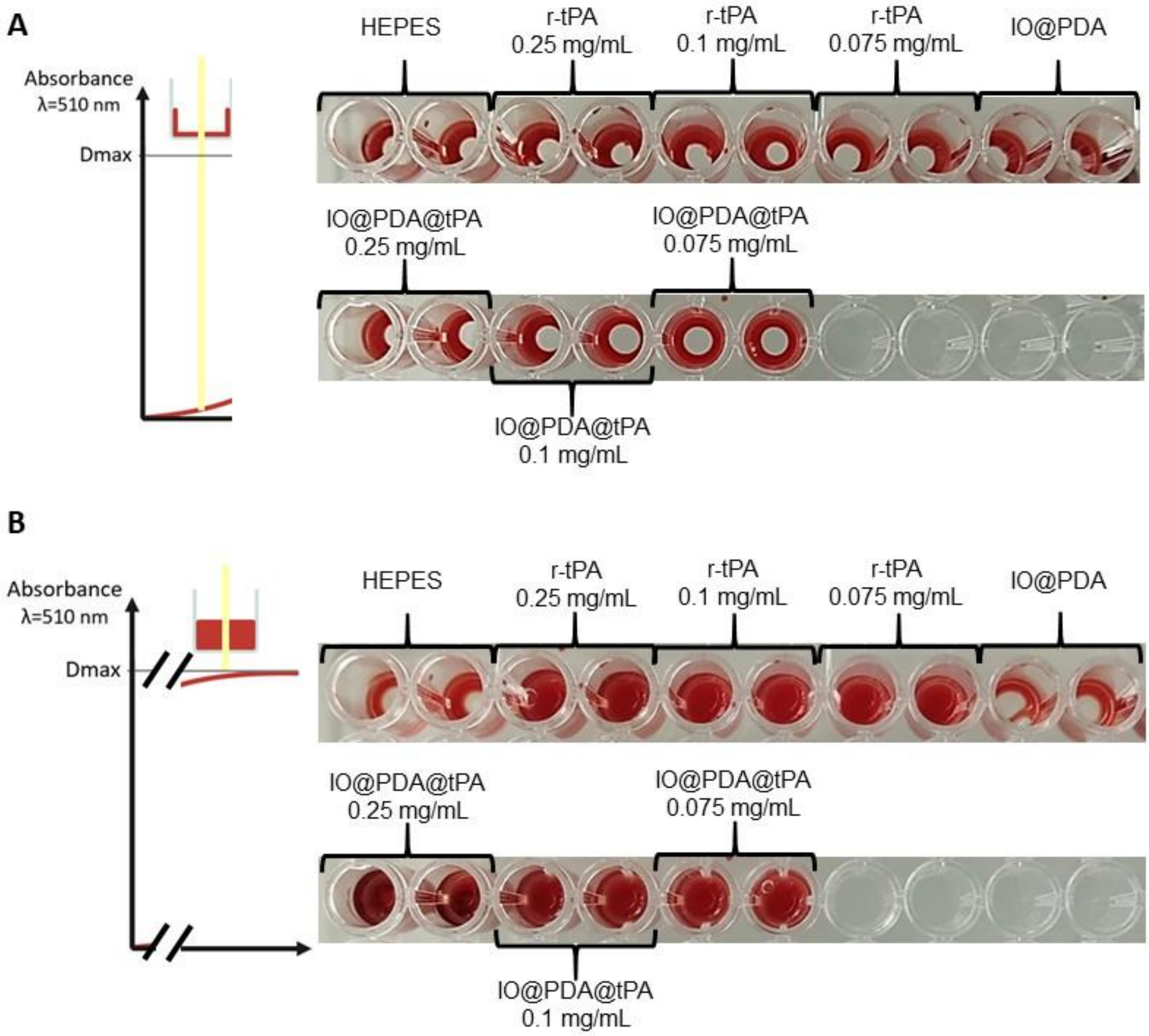

Supplementary Figure 3. Halo assay before and after treatment. Representative image of the halo assay clot after 30 min of incubation at 37 °C, immediately following treatment addition and just before starting the 2 h absorbance measurement (A). Representative image of the halo assay clot at the end of the 2 h absorbance acquisition (B).

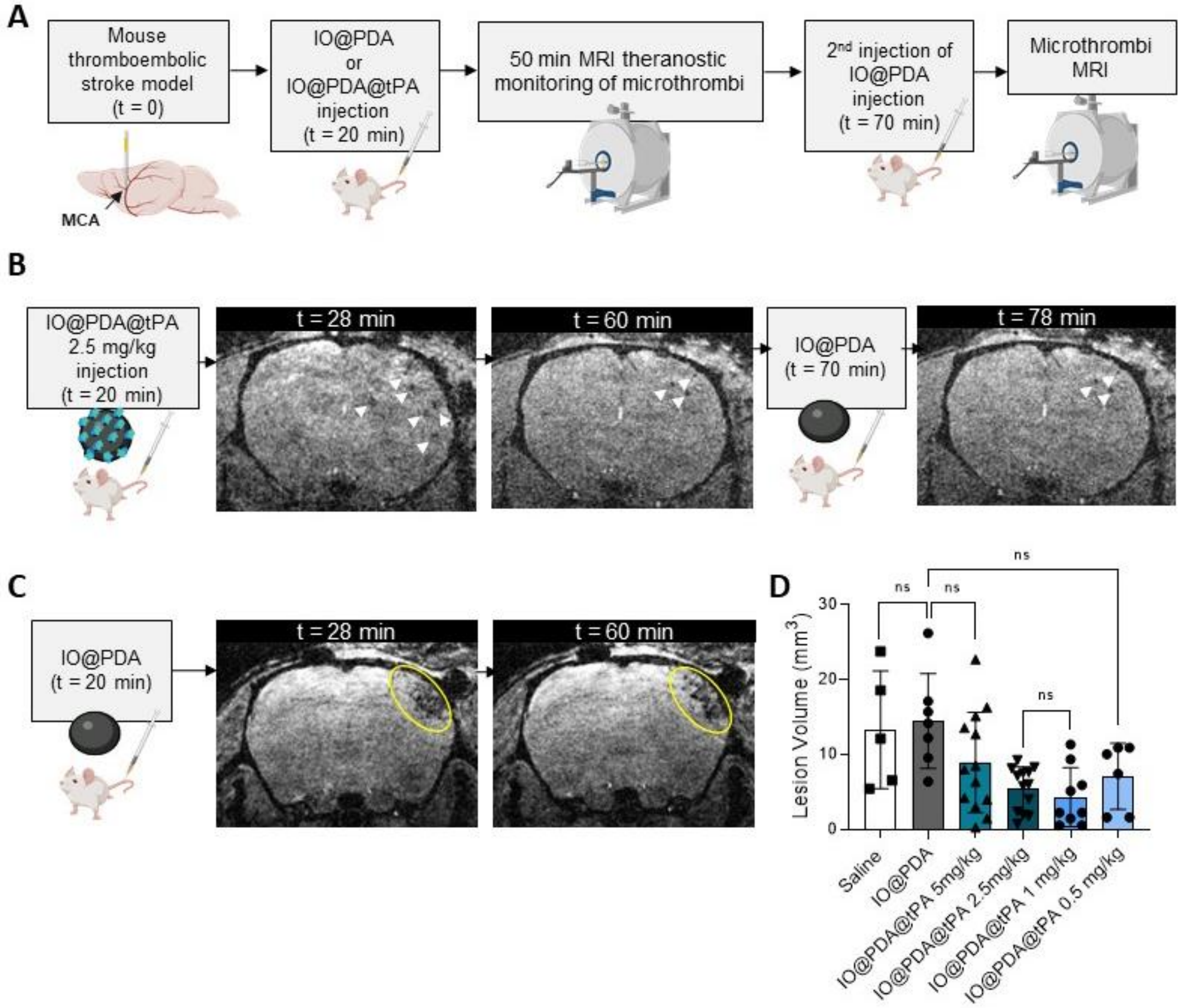

Supplementary Figure 4. Protocol illustration: IO@PDA@tPA injection after the thromboembolic stroke model followed by 1 h MRI acquisition and second injection of IO@PDA to reveal eventual remaining microthrombi (A). Injection of IO@PDA@tPA (2.5 mg/kg) revealed little amount of microthrombi. Further injection of IO@PDA after 60 min did not reveal any additional microthrombi (B). Injection of IO@PDA revealed microthrombi and no degradation was observed over 1 h (C). Lesion size of Saline condition and lower IO@PDA@tPA concentration (D).

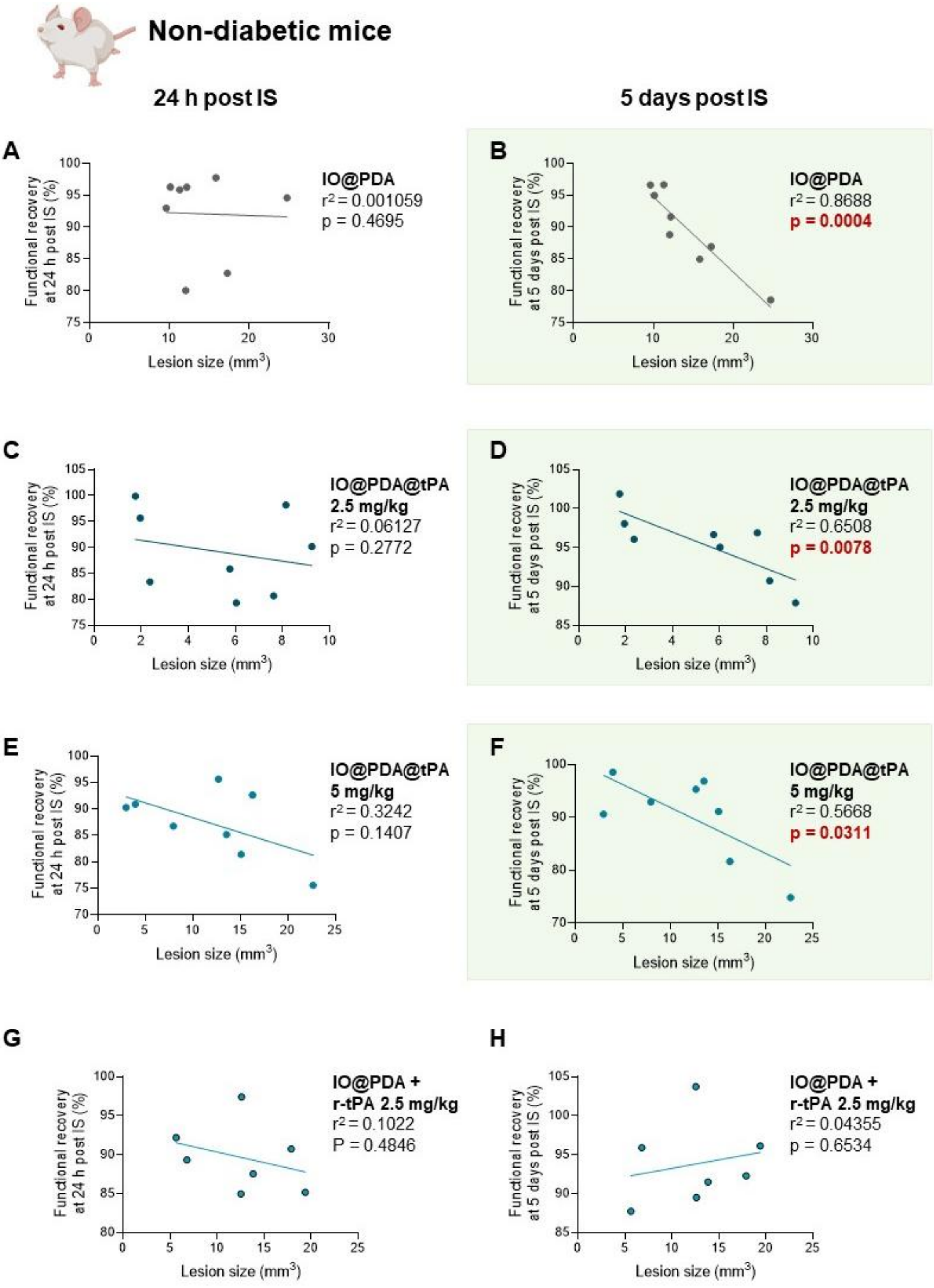

Supplementary Figure 5. Correlation between lesion size and functional recovery in non-diabetic mice. Correlation analyses were performed between lesion volume (mm^3) and functional recovery (%) at 24 h (A, C, E, G) and 5 days (B, D, F, H) after ischemic stroke. Treatments include IO@PDA (A–B), IO@PDA@tPA at 2.5 mg/kg (C–D) and 5 mg/kg (E–F), and IO@PDA combined with r-tPA at 2.5 mg/kg (G–H). Significant negative correlations were observed at 5 days for IO@PDA (B), IO@PDA@tPA 2.5 mg/kg (D), and IO@PDA@tPA 5 mg/kg (F), as highlighted by green panels.

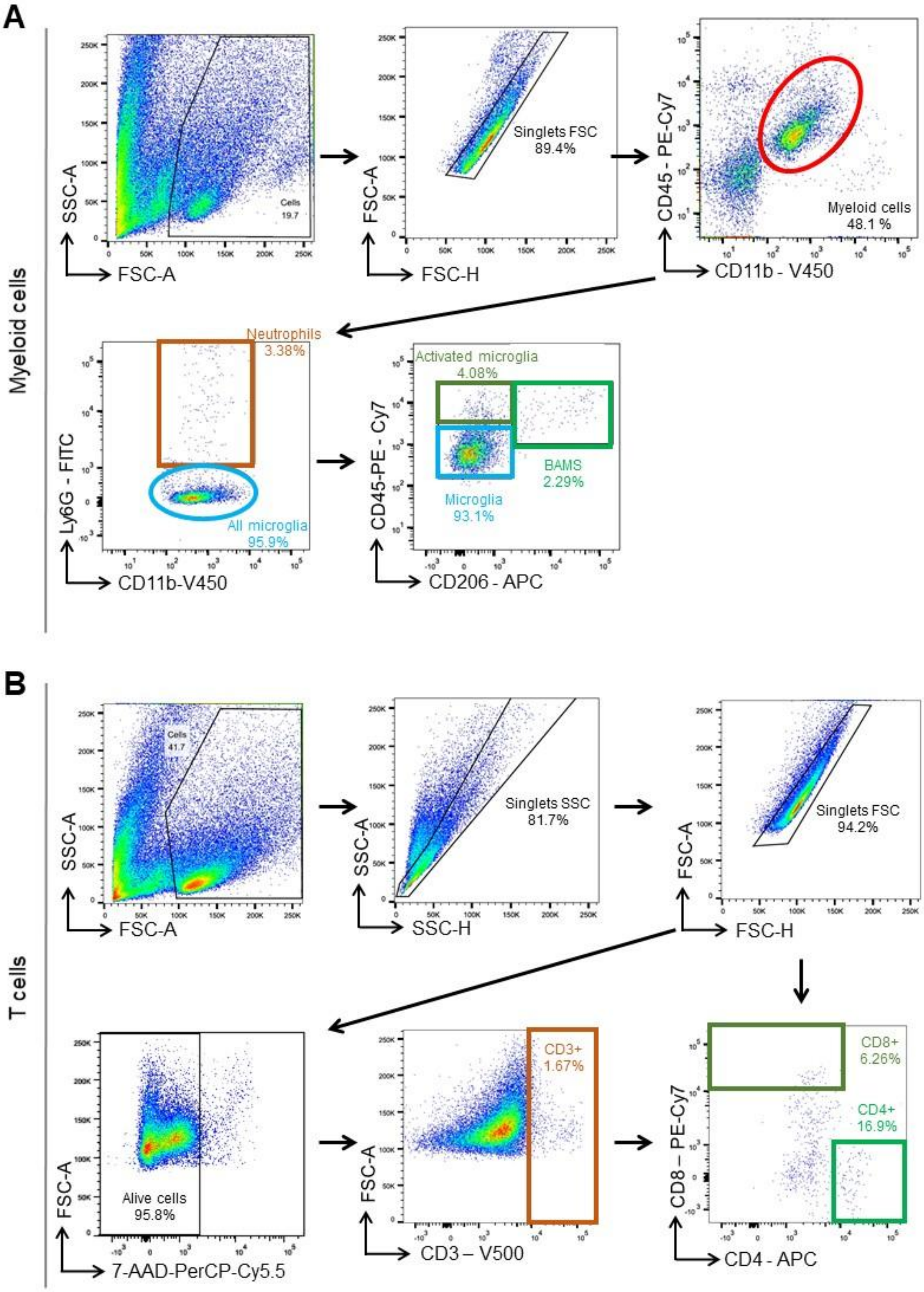

Supplementary Figure 6. Gating strategy for flow cytometry analysis of brain immune cell populations after IS. Gating strategy for quantification of brain myeloid and lymphoid cells. Single-cell suspensions were gated on cell granularity and size (SSC-A *vs* FSC-A), followed by doublet exclusion (FSC-A vs FSC-H). Myeloid cells were identified as $CD45^+$/$CD11b^+$. Neutrophils were defined as $CD11b^+$/$Ly6G^+$, while microglia were defined as $CD11b^+$/$Ly6G^-$. Among microglia, homeostatic microglia ($CD45^{int}$) and activated microglia ($CD45^{high}$) were distinguished based on the expression level of CD45, while CAMs were further distinguished based on CD206 expression (A). Single-cell suspensions were gated on cell granularity and size (SSC-A *vs* FSC-A), followed by a double doublet exclusion (SSC-A vs SSC-H and FSC-A vs FSC-H) as in (A). Alive cells (7-AAD-) were discriminated by the use of the 7-AAD. T lymphocytes were identified as $CD3^+$ cells. $CD8^+$ and $CD4^+$ T cells were distinguished using CD8 and CD4 surface markers (B).

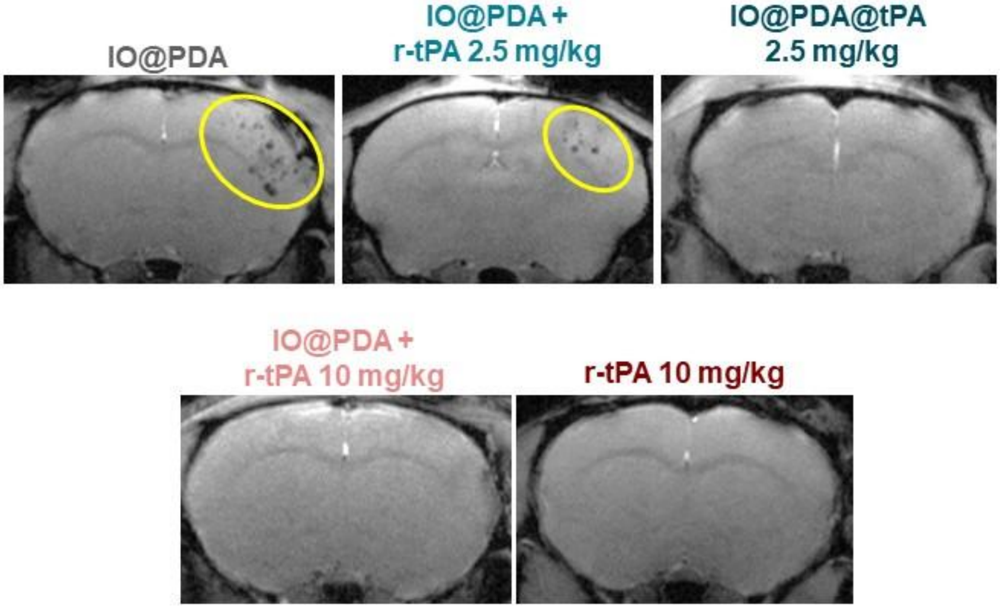

Supplementary Figure 7. T2s acquisitions revealed no hemorrhagic transformation at 24 h after stroke under any conditions. Nevertheless, a hypointense signal corresponding to the presence of microthrombi in the IO@PDA and IO@PDA + r-tPA 2.5 mg/kg was observed at 24 h after stroke. This highlighted that r-tPA at 2.5 mg/kg needed to be grafted on IO@PDA to be more efficient.

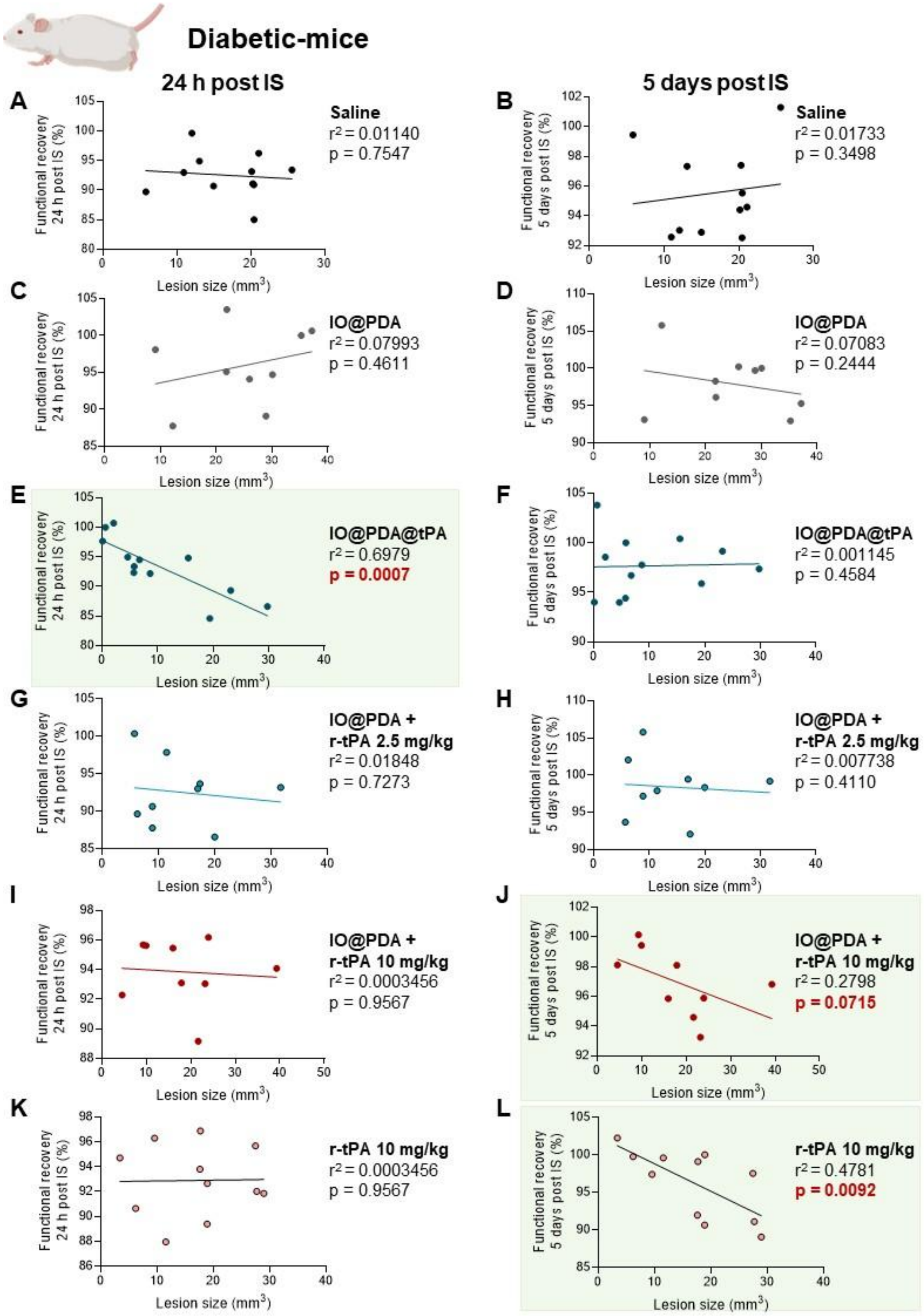

Supplementary Figure 8. Correlation between lesion size and functional recovery in diabetic mice. Correlation analyses were performed between lesion volume (mm³) and functional recovery (%) at 24 h (A, C, E, G, I, K) and 5 days (B, D, F, H,J,L) after ischemic stroke. Treatments include Saline (A–B), IO@PDA (C–D) and IO@PDA@tPA 2.5 mg/kg (E–F), IO@PDA combined with r-tPA at 2.5 mg/kg (G–H), IO@PDA combined with r-tPA at 10 mg/kg (I–J) and r-tPA at 10 mg/kg (K–L). Significant negative correlations were observed at 24h for IO@PDA@tPA 2.5 mg/kg (E) and at 5 days for IO@PDA + r-tPA 10 mg/kg (J), and r-tPA 10 mg/kg (L), as highlighted by green panels.

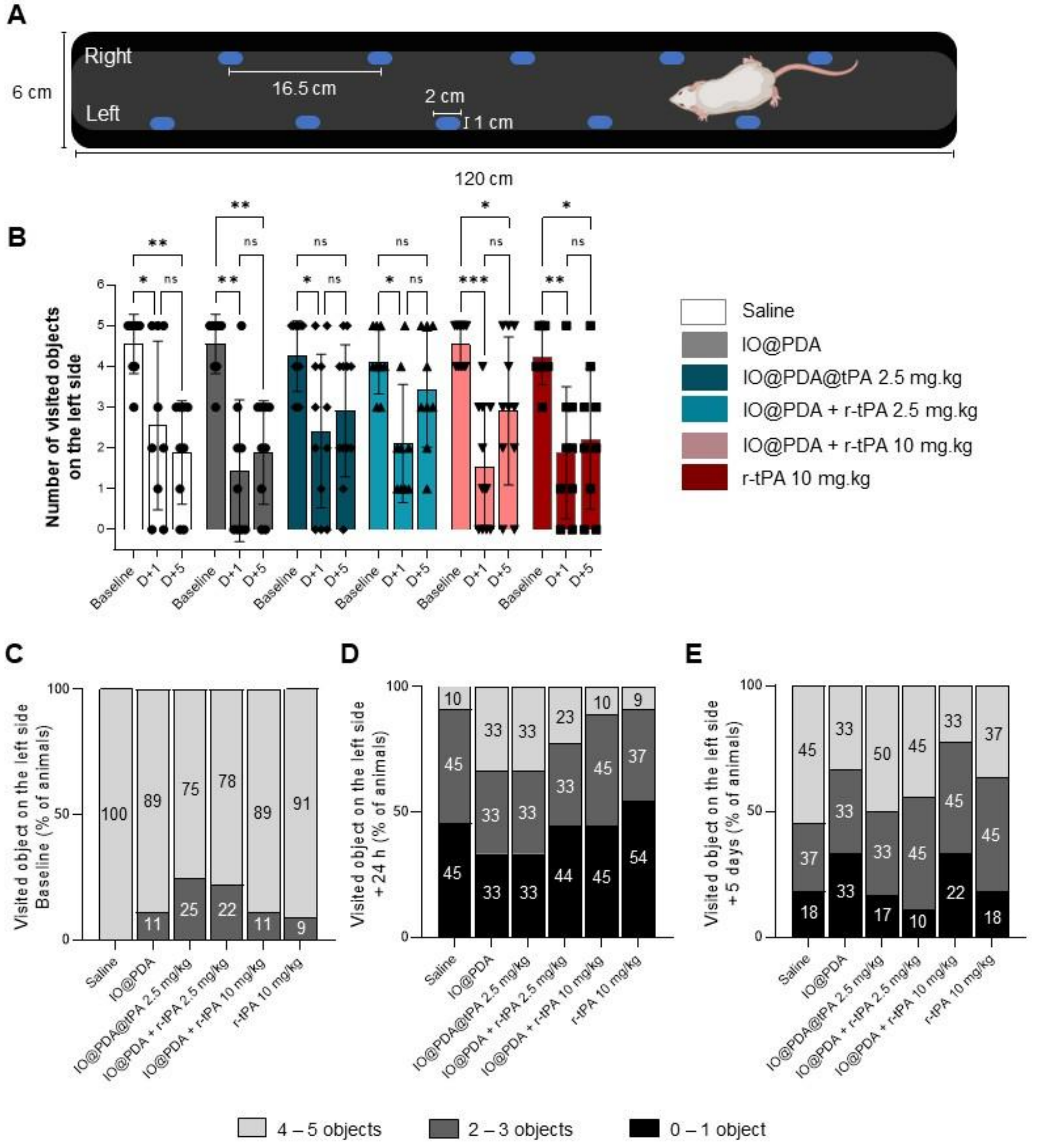

Supplementary figure 9. Mice treated with IO@PDA@tPA showed better sensorimotor recovery. Representation of the corridor device (A). Quantification of the visited objects on the left side by the mice treated with IO@PDA; IO@PDA@tPA 2.5 mg/kg; IO@PDA + r-tPA 2.5 mg/kg; r-tPA 10 mg/kg; IO@PDA + r-tPA 10 mg/kg before IS and at 1 and 5 days after IS (2way ANOVA, multiple comparisons, n = 9-12 per group) (B). More detail representation of the visited objects on the left side before IS (C), at 1 day (D) and 5 days after IS (E) (contingency table, n = 9-12). Data are represented as mean ± SD, *p < 0.05, **p < 0.01, ***p < 0.001, ****p < 0.0001, and ns = not significant, p > 0.05.
